# Intracellular Lipid Accumulation Drives the Differentiation of Decidual Polymorphonuclear Myeloid-Derived Suppressor Cells via Arachidonic Acid Metabolism

**DOI:** 10.3389/fimmu.2022.868669

**Published:** 2022-05-18

**Authors:** Qiaohong Wang, Xinyang Zhang, Congcong Li, Miao Xiong, Wenxin Bai, Si Sun, Chao Chen, Xiaoxin Zhang, Mingyang Li, Aimin Zhao

**Affiliations:** ^1^ Department of Obstetrics and Gynecology, Renji Hospital, School of Medicine, Shanghai Jiao Tong University, Shanghai, China; ^2^ Shanghai Key Laboratory of Gynecologic Oncology, Shanghai, China; ^3^ Department of Obstetrics and Gynecology, Shanghai Jiao Tong University School of Medicine Affiliated Sixth People’s Hospital, Shanghai, China

**Keywords:** polymorphonuclear myeloid-derived suppressor cells, immune tolerance in pregnancy, unexplained recurrent pregnancy loss, arachidonic acid metabolism, PGE2 synthesis, fatty acid-binding protein 5

## Abstract

Decidual polymorphonuclear myeloid-derived suppressor cells (PMN-MDSCs) are essential to immune tolerance during pregnancy. A reduction in the number of these cells is associated with unexplained recurrent pregnancy loss (URPL). In our previous study, we reported that PMN-MDSCs are a group of mature neutrophils that are activated by the decidua microenvironment. In the present study, we show that the decidua microenvironment induces substantial lipid accumulation in neutrophils during their differentiation to PMN-MDSCs. Lower levels of lipid accumulation are detected in PMN-MDSCs from URPL patients, and the amount of lipid in the PMN-MDSCs is positively correlated with the proportion of PMN-MDSCs. Next, we demonstrate that decidua-derived IL6 with the presence of arachidonic acid upregulates fatty acid-binding protein 5 (FABP5) *via* the phosphorylation of signal transducer and activator of transcription 3 (STAT3). Fy -60ABP5 then continuously stimulates intracellular lipid accumulation. Increased intracellular lipid accumulation mediates arachidonic acid metabolism, a pathway that is significantly activated by the induction of the decidua microenvironment, to stimulate the synthesis of prostaglandin E2 (PGE2) and finally induce the differentiation of PMN-MDSCs. To summarize, decidua-derived IL6 facilitates the differentiation of PMN-MDSCs from neutrophils *via* the pSTAT3/FABP5/PGE2 pathway. Defects in the process may result in impaired differentiation and dysfunction of PMN-MDSCs in URPL. These findings enhance our understanding of the physiological mechanisms of immune tolerance in pregnancy and provide therapeutic options for URPL.

## Introduction

Immunological tolerance is a key feature of human pregnancy, since the survival of the semi-allogeneic fetus needs to be protected against attack from the maternal immune system (1). Failure of maternal–fetal immune tolerance can lead to unexplained recurrent pregnancy loss (URPL) and other severe complications such as preeclampsia, fetal growth restriction, and preterm delivery (2–5). The maternal–fetal immune balance is jointly maintained by a rich variety of immune cells that are recruited during early pregnancy, including NK cells, T cells, macrophages, dendritic cells, and myeloid-derived suppressor cells (MDSCs) (2, 3, 6, 7). Decidual MDSCs play an essential role during pregnancy (7–10), as well as creating a complicated network with other immune cells (11–14). The abnormal expansion and accumulation of MDSCs are associated with URPL and other pregnant complications (15–18).

MDSCs are highly immunosuppressive populations and can be divided into polymorphonuclear MDSCs (PMN-MDSCs) and monocytic MDSCs (M-MDSCs) (19). PMN-MDSCs are the dominant subgroup of MDSCs in human decidua during early pregnancy (11, 13, 14, 20). PMN-MDSCs have morphological and phenotypic features that are similar to peripheral neutrophils. Because of the high plasticity of neutrophils (21–23), tissue-resident PMN-MDSCs are able to differentiate from neutrophils (20, 24, 25). In our previous study, we determined the mature and activated states of decidual PMN-MDSCs and reported that decidual PMN-MDSCs are a group of mature and activated neutrophils that are induced to differentiate by the decidua microenvironment (20). However, the mechanism of differentiation that underlies this transition has yet to be elucidated.

The phenotypic and functional heterogeneity of MDSCs can be regulated by metabolic and inflammatory factors (26, 27). The complex interactive network created by the tumor microenvironment stimulates the metabolic reprogramming of MDSCs, thus driving their differentiation and function (28–30). Lipid metabolic reprogramming is preferentially utilized in non-inflammatory and tolerogenic immune cells, including T regulatory cells, M2 macrophages, and MDSCs (9, 31, 32). Lipid is important for maintaining cell membrane integrity, homeostasis, signal transduction, and other biological functions. Recent studies provide evidence that lipid is also implicated in the accumulation and functional regulation of MDSCs (33–35). Lipid accumulation in tumor-infiltrating MDSCs is known to promote their immunosuppressive activity (27, 36, 37). The upregulation of fatty acid transportation (38, 39) and fatty acid oxidization (27, 36), as well as substantial lipid accumulation (36–38), have all been associated with the enhanced expansion and function of tumor-derived MDSCs. Hypoxia and immune stimuli can result in metabolic reprograming (40), since normal pregnancy can also be regarded as a microenvironment that involves hypoxia and chronic inflammation, especially during early pregnancy. However, insights from the driving effect of lipid on the differentiation and function of MDSCs have yet to stimulate research in the context of pregnancy.

In the present study, we investigated the potential effect of lipid accumulation on the differentiation of PMN-MDSCs in a cohort of healthy women during early pregnancy. We found that decidua-derived interleukin-6 (IL6) promoted fatty acid transport and lipid accumulation in the presence of arachidonic acid (AA) by regulating the phosphorylation of signal transducer and activator of transcription 3 (STAT3) and the expression of fatty acid-binding receptor 5 (FABP5). Intracellular lipid and subsequent AA metabolisms were then able to manipulate the differentiation of decidual PMN-MDSCs from circulating neutrophils. This information enhances our understanding of the potential mechanisms of impaired PMN-MDSC differentiation in URPL and provides options for therapeutic intervention.

## Materials and Methods

### Participants

Decidua tissues and autologous peripheral blood samples were provided by healthy women in early pregnancy (n = 61) who underwent elective surgical pregnancy termination for non-medical reasons at the Department of Obstetrics and Gynecology, Renji Hospital, School of Medicine, Shanghai Jiao Tong University. The clinical characteristics of the participants are listed in **Supplementary Table S1**. Twenty-one patients with URPL were also enrolled in the study. The characteristics of URPL patients, and a comparison with the normal pregnancy (NP) group, are given in **Supplemental Table S2**. Misoprostol and mifepristone were not administered to either group. A fetal heartbeat in the NP group was confirmed by ultrasound prior to the elective termination of pregnancy. Patients in the URPL group met the following criteria (1): two or more previous pregnancy losses (2); fetal heartbeat had ceased or was never detected (3); normal karyotype in both parents and the abortus; and (4) no evidence for other risk factors, including uterine malformation, infection, thrombophilia, or autoimmune, endocrine, or metabolic diseases. Samples of endometrium and decidua tissue samples were collected to obtain explant supernatant for cytokine and AA analysis or neutrophil stimulation. Endometrium tissues were obtained from non-pregnant women undergoing surgery for benign diseases during the secretory phase at Renji Hospital. Peripheral blood for the isolation of T cells or neutrophils was collected from 40 healthy non-pregnant donors. All participants signed the informed consent, and the study was approved by the Human Research Ethics Committee of Renji Hospital.

### Cell Preparation

Decidual PMN-MDSCs were isolated as previously described (16, 20). In brief, decidua tissues were mechanically dissociated by a gentleMACS Dissociator (Miltenyi Biotec, Auburn, CA, USA) and passed through a 70-μm strainer and then a 40-μm strainer. Density gradient centrifugation and CD15 MicroBeads (Miltenyi Biotec) were used to purify the decidual PMN-MDSCs. Peripheral blood mononuclear cells (PBMCs) and neutrophils were obtained by density gradient centrifugation and the lysis of red blood cells. Peripheral neutrophils were then purified using CD15 MicroBeads, while CD3^+^ T cells were isolated from PBMCs using CD3 MicroBeads (Miltenyi Biotec).

### Preparation of Tissue Explant Supernatant

Tissue explant supernatant was collected as previously described (20, 41, 42). Decidua tissues and endometrial tissues (1 × 1 × 0.3 cm) were placed in 24-well plates, containing 1 ml of RPMI-1640 (HyClone, South Logan, UT, USA) supplemented with 10% fetal bovine serum (FBS; Gibco BRL, Grand Island, NY, USA) and 1% penicillin/streptomycin/amphotericin B (Gibco BRL). After a 24-h incubation at 37°C in a humidified atmosphere containing 5% CO_2_, we collected decidual explant supernatant (DES) and endometrial explant supernatant (EES) by centrifugation at 3,000g for 10 min. The supernatants were then stored at -80°C.

### Neutrophil Stimulation

Neutrophils from healthy donors were stimulated with 50% (v/v) DES, 50% (v/v) EES, recombinant human IL6 (100 ng/ml, R&D Systems, Minneapolis, MN, USA), recombinant human GM-CSF (100 ng/ml; R&D Systems), G-CSF (100 ng/ml; R&D Systems), and recombinant human IL-8 (100 ng/ml; R&D Systems) supplemented with AA (10 μM; MedChemExpress, Monmouth Junction, NJ, USA) for 16 h. For signaling pathway inhibition, neutrophils were pretreated with a STAT3 inhibitor (Stattic, 10 μM; Selleck Chemicals, Houston, TX, USA) or FABP5 inhibitor (SBFI26, 200 μM; MedChemExpress) for 2 h. Then, the cells were treated with 50% (v/v) DES or IL6 supplemented with AA for 16 h. After stimulation, the cells were harvested for flow cytometry or functional analysis.

### Quantitative Real-Time PCR

Total RNA was extracted from isolated decidual PMN-MDSCs and neutrophils using TRIzol (Invitrogen, Carlsbad, CA, USA). cDNA was synthesized using a PrimeScript RT Reagent Kit (Takara, Tokyo, Japan) in accordance with the manufacturer’s instructions. Quantitative real-time PCR analysis was performed using SYBR Premix Ex Taq II (Takara) with a QuantStudio Dx Real-Time Instrument (Life Technologies, Gaithersburg, MD, USA). The relative gene expression was calculated using the 2^−ΔCT^ method for clinical samples and the 2^−ΔΔCT^ method for other situations. Primer sequences are given in **Supplementary Table S3**.

### Flow Cytometry

An Fc receptor blocking solution (BioLegend, San Diego, CA, USA) was added prior to staining with the following antibodies: CD45, CD11b, CD66b, pSTAT3 (BD Biosciences, San Jose, CA, USA), CD33, HLA-DR, CD15, CD3, CD4, CD8, arginase 1 (ARG1), IL6Rα, IFN-γ (BioLegend), inducible nitric oxide synthase (iNOS) (Santa Cruz Biotechnology, Santa Cruz, CA, USA), and FABP5 (R&D Systems). The mouse IgG1 and rat IgG2a isotype controls were used (BioLegend). Intracellular staining was performed using a Fixation/Permeabilization Kit (BD Biosciences) and a Transcription Factor Phospho Buffer Set (BD Biosciences). Reactive oxygen species (ROS) were detected using a CellROX Green Flow Cytometry Assay Kit (Thermo Fisher Scientific, Waltham, MA, USA) in accordance with the manufacturer’s instructions. To quantify lipid accumulation by flow cytometry, cells were first stained with surface markers and then with BODIPY 493/503 (250 ng/ml; Thermo Fisher Scientific) in 500 μl of PBS for 15 min at room temperature. To test the uptake of free fatty acids, cells were stained with BODIPY FL C16 (1μM; Thermo Fisher Scientific) for 30 min at 37°C. Flow cytometry was performed with an LSR Fortessa (BD Biosciences), and data were analyzed using FlowJo software (BD Biosciences). Fluorescent minus one (FMO), along with an isotype control, was used to set up appropriate gates. The mean fluorescent intensity (MFI) using the arithmetic mean was used in subsequent analyses.

### Immunofluorescence

To stain lipid by immunofluorescence, we incubated cells with BODIPY 493/503 (2 μM) for 15 min at 37°C in the dark. Then, the cells were fixed in 4% paraformaldehyde for 30 min at room temperature and stained with 4, 6-diamidino-2-phenylindole (DAPI, BD Biosciences) for the detection of cell nuclei.

### T-Cell Suppression Assay

CD3^+^ T cells were isolated from PBMCs by CD3 microbeads. T cells were labeled with CFSE (BD Biosciences) and activated with precoated anti-CD3 (10 μg/ml; OKT3, BioLegend) and soluble anti-CD28 (1 μg/ml; CD28.2, BioLegend) and then cocultured with neutrophils (with or without stimulation) at a ratio of 2:1. The proliferation of T cells was then assessed after 3.5 days. For T-cell secretion suppression assay, we used unlabeled T cells in the coculture system. Before the cells were harvested for the analysis of intracellular IFN-γ expression, a leukocyte activation cocktail (BD Biosciences) was added into the culture system for 5 h.

### Enzyme-Linked Immunosorbent Assay

Enzyme-linked immunosorbent assay (ELISA) kits for AA (FineTest, Wuhan, China) and IL6 (MultiSciences Biotech, Hangzhou, China) were used to detect the specific levels of AA and IL6 in DES and EES in accordance with the manufacturer’s instructions. We also measured the levels of prostaglandin E2 (PGE2) (MultiSciences Biotech) levels, in cell supernatants derived from decidual PMN-MDSCs and neutrophils.

### Gene Expression Microarray and Transcriptome Sequencing

Decidual PMN-MDSCs and autologous neutrophils (n = 3) were treated with TRIzol (Invitrogen) for the extraction of total RNA. An Agilent SurePrint G3 Human Gene Expression v3 8x60K Microarray (DesignID:072363) was used. The arrays were then scanned using an Agilent Scanner G2505C (Agilent Technologies, Santa Clara, CA, USA) conducted by OE Biotechnology Co., Ltd. (Shanghai, China). Gene set enrichment analysis (GSEA) was carried out in the R environment. The complete dataset was deposited in the Gene Expression Omnibus (GEO) database of the National Center for Biotechnology Information (NCBI); the accession number was GSE192850. We also extracted total RNA from neutrophils that had been treated with or without 50% (v/v) DES (n = 3). Transcriptome libraries were generated with an Illumina TruSeq™ RNA Sample Prep Kit (Illumina, San Diego, CA, USA) and sequenced on an Illumina NovaSeq 6000 platform by Majorbio Bio-Pharm Technology Co., Ltd. (Shanghai, China). Finally, the data were analyzed on the free online platform provided by Majorbio Cloud Platform and were also deposited in the GEO database; the accession number was GSE193491.

### Statistical Analysis

All analyses were performed using Prism version 9 Software (GraphPad Software, San Diego, CA, USA). Data are presented as mean ± standard deviation (SD). Differences between two groups were analyzed with the Student’s *t*-test. One-way analysis of variance (ANOVA), followed by Tukey’s *post-hoc* test, was used to analyze the differences between multiple groups. Correlations between parameters were evaluated by Pearson correlation analysis. Significance was defined as *p < 0.05, **p < 0.01, ***p < 0.001, and ****p < 0.0001.

## 3 Results

### An Increased Lipid Content in Decidual PMN-MDSCs Which Was Correlated With an Increased Proportion

**Figure 1:**
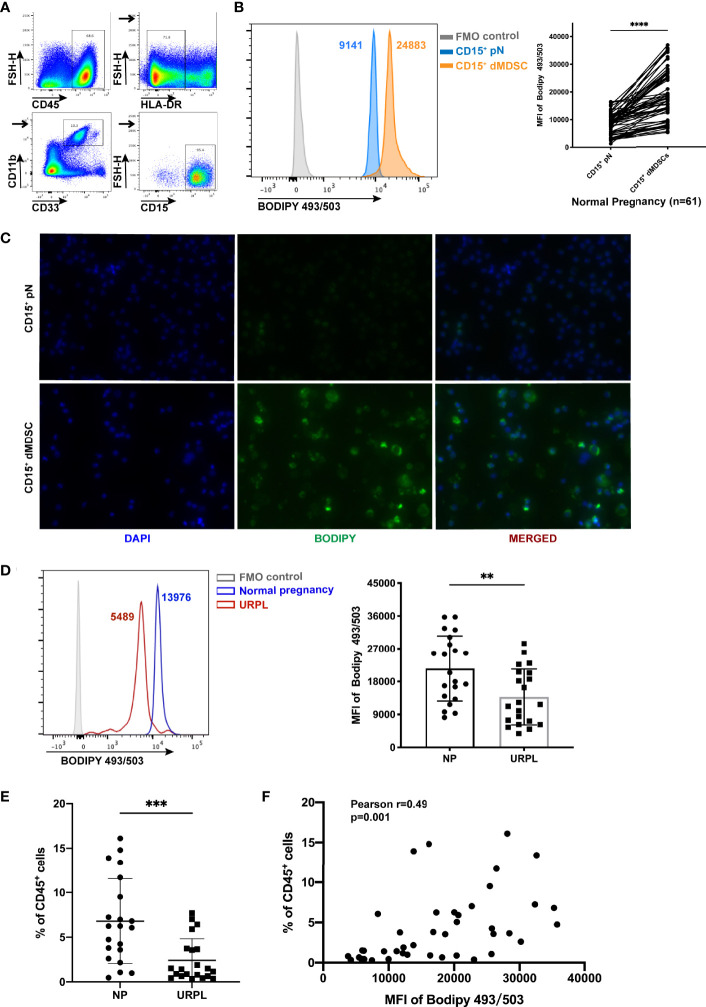
Increased lipid accumulation in decidual polymorphonuclear myeloid-derived suppressor cells (PMN-MDSCs). **(A)** Flow cytometry showing the gating strategy for PMN-MDSCs (gated on CD45+HLA-DR-/lowCD11B+CD33dimCD15+CD14-). **(B)** Lipid accumulation of CD15+ decidual MDSCs (dMDSCs) and autologous peripheral neutrophils (pN) (n = 61) in normal pregnancy (NP). **(C)** Immunofluorescence images of BODIPY 493/503 (green) in CD15+ dMDSCs and autologous pN. **(D)** Lipid accumulation in CD15+ dMDSCs in NP (n = 21) and URPL (n = 21) patients. **(E)** The proportion of CD15+ dMDSCs was compared between NP (n = 21) and unexplained recurrent spontaneous abortion (URPL) (n = 21). **(F)** The correlation of lipid accumulation in CD15+ dMDSCs with the proportion of CD15+ dMDSCs within a population of CD45+ leukocytes (n = 42). All data are represented as mean ± SD. Significance was determined by paired or unpaired Student’s t-test. ****p < 0.0001, ***p < 0.001, **p < 0.01. FMO, fluorescent minus one.

To evaluate the total lipid content in decidual PMN-MDSCs, identified as CD45^+^HLA-DR^-/low^CD11B^+^CD33^dim^CD15^+^CD14^-^ (**Figure 1A**), and circulating CD15^+^ neutrophils, we detected the staining of BODIPY 493/503 by flow cytometry and immunofluorescence (**Figures 1B, C** and **Figure S1**). Decidual PMN-MDSCs (dMDSCs) contained a considerably larger amount of lipid than autologous peripheral neutrophils (pN) from women in early pregnancy (n = 61, *p* < 0.0001). However, the accumulation of lipid was remarkably reduced in URPL patients (**Figure 1D**; *p* = 0.0042). Known as the most abundant subset of MDSCs subset in the decidua, PMN-MDSCs were reduced in URPL patients when compared to normal pregnancy (**Figure 1E**) (16). Further analysis revealed that the proportion of decidual PMN-MDSCs was positively correlated with intracellular lipid accumulation (**Figure 1F**; Pearson r = 0.49, *p* = 0.001).

### Fatty Acid Transmembrane Transporter Activity and Fatty Acid Metabolism Pathways Were Upregulated in Decidual PMN-MDSCs

**Figure 2:**
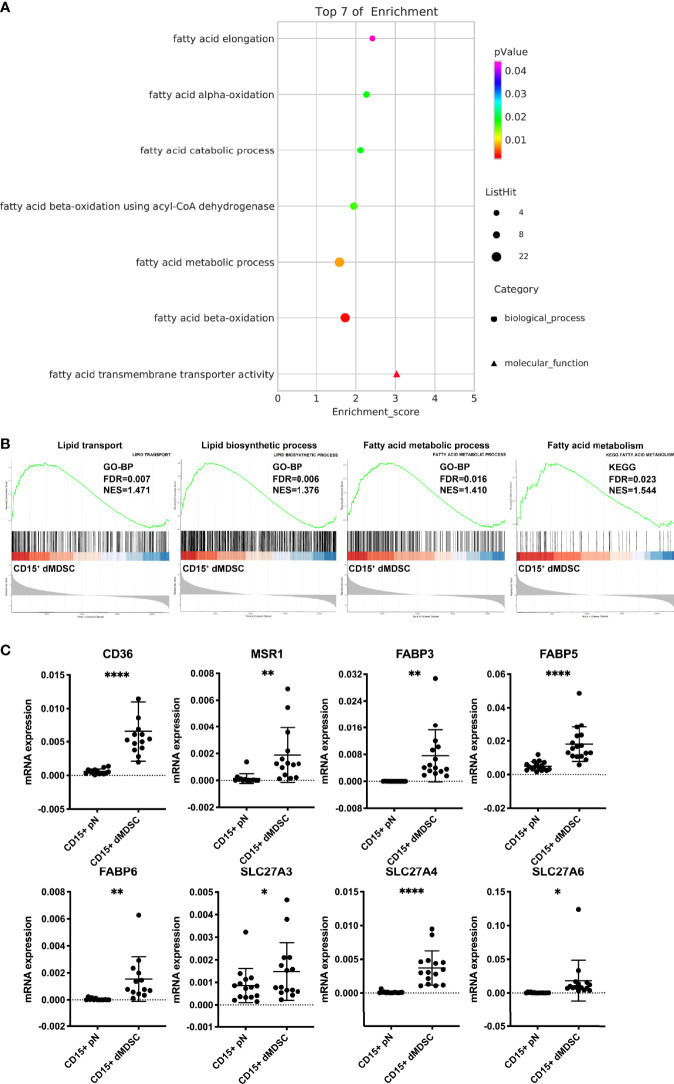
Upregulation of fatty acid transmembrane transporter activity in decidual PMN-MDSCs. **(A)** GO enrichment analysis of lipid-related pathways in decidual PMN-MDSCs and autologous neutrophils in healthy women during early pregnancy (n = 3). **(B)** GSEA showed that gene sets related to lipid metabolism were highly enriched in CD15+ dMDSCs, including lipid transport, lipid biosynthetic process, fatty acid metabolic process (GO term), and fatty acid metabolism (KEGG pathway). **(C)** The differential expression of lipid transport receptors was detected between CD15+ dMDSCs and autologous pN in NP, including CD36, MSR1, FABP3, FABP5, FABP6, SLC27A3, SLC27A4, SLC27A5, and SLC27A6. All data are presented as mean ± SD. Significance was determined by unpaired Student’s t-test. ****p < 0.0001, **p < 0.01, *p < 0.05.

Next, we performed whole-genome expression profile analysis for decidual PMN-MDSCs and autologous neutrophils. By performing Gene Ontology (GO) analysis with a threshold criteria of *p* < 0.05, we identified seven lipid-related pathways that were significantly upregulated in decidual PMN-MDSCs, thus suggesting that multiple biological processes are responsible for the differential lipid levels between decidual PMN-MDSCs and circulating neutrophils (**Figure 2A**). The fatty acid transmembrane transporter activity pathway was the most significantly enriched pathway. GSEA indicated that several gene sets, including lipid transport, lipid biosynthetic process, and fatty acid metabolic process (GO term), along with fatty acid metabolism (KEGG pathway), were significantly enriched in decidual PMN-MDSCs (**Figure 2B**). We also detected a number of lipid transport receptors that are implicated in fatty acid uptake and lipid trafficking, including CD36, MSR1, fatty acid-binding proteins (FABPs), and solute carrier family member 27 (SLC27; also known as fatty acid transport proteins, FATPs) (43). We also identified increased mRNA levels of *CD36*, *MSR1*, *FABP3*, *FABP5*, *FABP6*, *SLC27A3*, *SLC27A4*, and *SLC27A6* in decidual PMN-MDSCs (**Figure 2C**; **Figure S2**).

### Decidua-Derived Factors Promoted Lipid Accumulation and Fatty Acid Metabolism in Neutrophils

**Figure 3:**
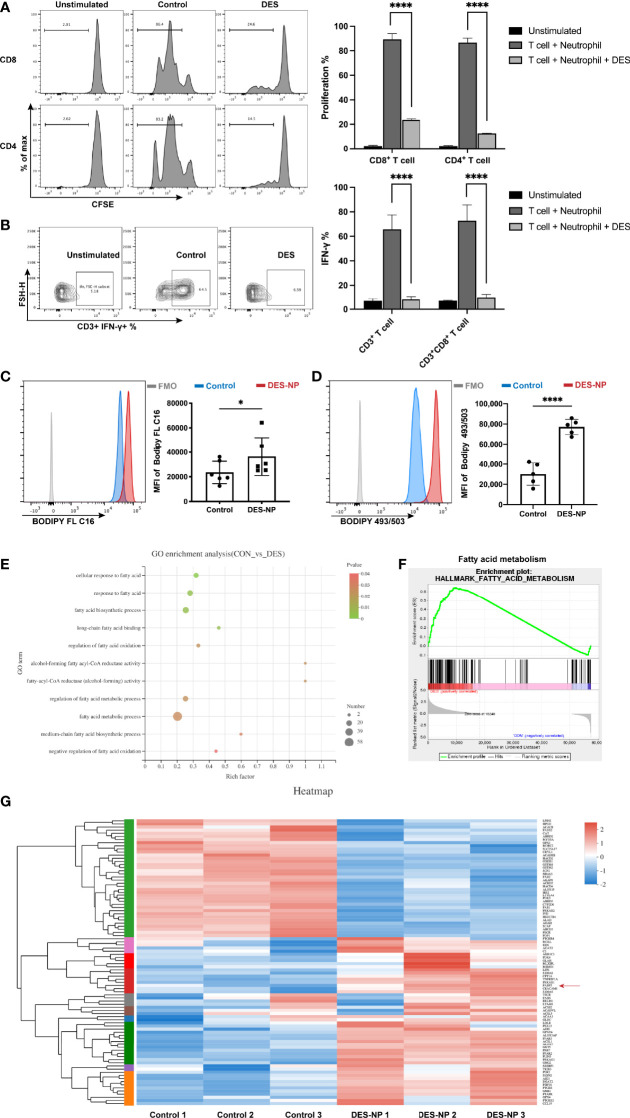
Induced lipid accumulation and the enrichment of fatty acid metabolism after exposure to decidual explant supernatant (DES). To evaluate the functional characteristics of DES-conditioned circulating neutrophils, CD3/CD28-stimulated T cells were cocultured with circulating neutrophils from healthy donors either untreated or treated with 50% (v/v) DES at a ratio of 2:1 for 3.5 days. The proportion of proliferative CD4+ T cells or CD8+ T cells (n = 6) **(A)** and the percentage of IFN-γ-expressing CD3+ T cells or CD8+ T cells (n = 6) **(B)** were analyzed. **(C, D)** Increased free fatty acid uptake (BODIPY FL C16) (n = 6) and intracellular lipid accumulation (BODIPY 493/503) (n = 5) were detected after exposure to DES. **(E)** GO terms associated with fatty acid metabolism in circulating neutrophils were upregulated after DES treatment (n = 3). **(F)** GSEA showing enrichment of the fatty acid metabolism gene set in the DES-condition. **(G)** Heatmap showing the differentially expressed genes related to lipid metabolism. All data are represented as mean ± SD. Significance was determined by the unpaired Student’s t-test. ****p < 0.0001, *p < 0.05. FMO, fluorescent minus one.

We recently reported that the decidua microenvironment can stimulate circulating CD15^+^ neutrophils to develop a PMN-MDSC-like phenotype and function (20). In the present study, we demonstrated that after treatment with DES, CD15^+^ neutrophils significantly suppressed the proliferation of both CD4^+^ T cells and CD8^+^ T cells (**Figure 3A**). IFN-γ secretion was also suppressed by DES-conditioned neutrophils (**Figure 3B**). This induced T-cell suppression ability is a critical characteristic and implies that the circulating CD15^+^ neutrophils had gained MDSC-defined functionality and differentiated into MDSCs. Furthermore, we detected increased free fatty acid uptake (**Figure 3C**) and intracellular lipid (**Figure 3D**) in DES-conditioned neutrophils, as evidenced by respectively staining with BODIPY FL C16 and BODIPY 493/503. DES facilitated immune tolerance in circulating neutrophils; this was accompanied by a substantial amount of lipid accumulation.

Transcriptomic sequencing of neutrophils treated or untreated with 50% (v/v) DES revealed the upregulation of several pathways relating to fatty acid metabolism (as GO terms) after induction with DES (**Figure 3E**). GSEA showed that gene sets associated with fatty acid metabolism were highly enriched under DES conditions (**Figure 3F**). A heatmap was generated to visualize the differentially expressed genes relating to lipid metabolism (**Figure 3G**). *S100A8* and *S100A9*, transcription factors that are known to induce the differentiation of MDSCs, were upregulated after DES treatment. In addition, sterol regulatory element-binding protein 1 (*SREBF1*) and associated genes downstream [fatty acid synthase (*FASN*) and acetyl-CoA synthetase 2 (*ACSS2*)] were upregulated, thus suggesting that DES induced the activation of fatty acid biosynthesis and metabolic reprograming in neutrophils. The upregulation of carnitine palmitoyltransferase 1A (*CPT1A*), a gene that encodes the rate-limiting enzyme for fatty acid oxidation, suggested that DES might induce fatty acid oxidation and promote the utilization of lipids. Most importantly, of all the fatty acid receptors, only *FABP5* showed significant upregulation following induction by DES (**Figure 3G**). This suggested that the increased lipid uptake and accumulation induced by the decidua microenvironment might drive fatty acid metabolism and thus promote differentiation into PMN-MDSCs.

### IL6 Derived From the Decidua Promoted Intracellular Lipid Accumulation With the Supplementation of Arachidonic Acid

**Figure 4:**
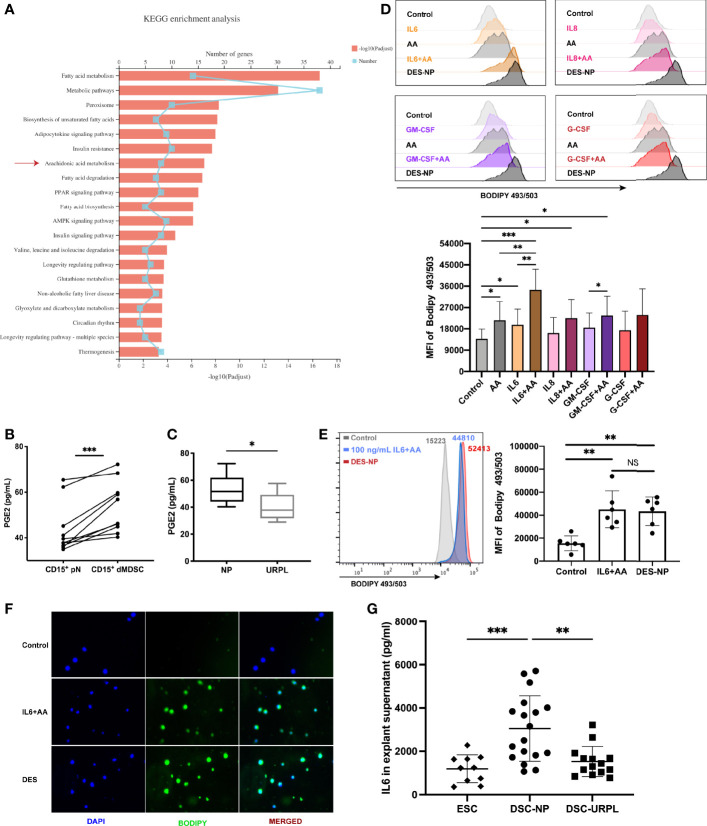
The promotional effect of IL6, a cytokine derived from the decidua, on lipid accumulation supplemented with arachidonic acid (AA). **(A)** KEGG enrichment analysis of differentially expressed genes in pathways related to fatty acid metabolism. **(B)** The secretion of PGE2 in CD15+ dMDSCs and autologous pN was detected by ELISA (n = 10). **(C)** A difference in PGE2 secretion from PMN-MDSCs was found between NP (n = 10) and URPL (n = 5). **(D)** The effects of different decidual-derived cytokines on lipid accumulation in neutrophils were evaluated by flow cytometry, including IL6, IL8, GM-CSF, and G-CSF (n = 5). **(E)** IL6 promoted the utilization of AA by circulating neutrophils, thus resulting in a similar effect to DES (n = 6). **(F)** Representative immunofluorescence images of BODIPY 493/503 (green) in neutrophils treated with IL6+AA or DES. **(G)** Differences in IL6 secretion between EES (n = 10), DES-NP (n = 15), and DES-URPL (n = 14). All data are represented as mean ± SD. Significance was determined by paired or unpaired Student’s t-test, or one-way ANOVA with Tukey’s post-hoc test. ***p < 0.001, **p < 0.01, *p < 0.05, NS, not significant. FMO, fluorescent minus one.

Kyoto Encyclopedia of Genes and Genomes (KEGG) pathway analysis revealed the top 20 enriched fatty acid metabolism pathways; of these, the arachidonic acid metabolism pathway was found to be a very important and differential pathway (**Figure 4A**). AA is a polyunsaturated fatty acid that serves a range of important biochemical roles, including acting as the direct precursor of PGE2 (44). PGE2 is a key molecule in the biology of MDSCs, particularly with regard to the induction of immunosuppressive activity in MDSCs (45–48). Using ELISA, we confirmed that PMN-MDSCs released significantly larger amounts of PGE2 than autologous neutrophils (**Figure 4B**). Moreover, we found a significant reduction in the secretion of PGE2 from PMN-MDSCs isolated from URPL patients (**Figure 4C**), thus suggesting that decidual PMN-MDSCs are dysfunctional in URPL patients. We detected upregulated levels of lipid accumulation in neutrophils when AA was supplemented in the cultivation system; however, there was a dramatic difference between AA-conditioned and DES-conditioned cells (**Figure S3A**). Furthermore, we found that EES when compared to DES did not induce a statistically significant increase in intracellular lipid accumulation (**Figure S3B**). In addition, there was no significant difference in the levels of AA between the endometrium and the decidua (**Figure S3C**). Similarly, no significant difference was detected between NP and URPL decidua (**Figure S3D**). Collectively, these findings support the hypothesis that the presence of soluble cytokines in DES can continuously stimulate lipid accumulation in neutrophils by promoting the uptake of AA.

In our previous study, we used Luminex assay to detect the expression levels of 27 cytokines and found that several cytokines, but particularly IL6, IL8, GM-CSF, and G-CSF, were highly and differentially expressed in DES and EES (20). In the present study, only IL6 was found to promote AA-related lipid synthesis (**Figures 4D**, **S3E**). There was no difference between the AA-related lipid accumulation promoted by IL6 and that induced by DES (**Figure 4E**). Immunofluorescence assay also exhibited a visualized increase in BODIPY 493/503 expression after IL6+AA treatment (green; **Figure 4F**). Furthermore, ELISA verified that the levels of IL6 in DES-NP were notably higher than that in EES; the levels of IL6 were significantly lower in DES-URPL than in DES-NP (**Figure 4G**). We also verified that both decidual PMN-MDSCs and circulating CD15^+^ neutrophils expressed the IL6 receptor in a stable manner (IL6Rα) (**Figure S3F**). Collectively, these results highlight the role of decidua-derived IL6 in promoting lipid accumulation in neutrophils with AA supplementation and suggest a potential role for the impaired differentiation of PMN-MDSCs in URPL patients.

### IL6 Promoted the Expression of Immune-Regulatory Molecules and Facilitated Immune Tolerance *via* Arachidonic Acid Metabolism

**Figure 5:**
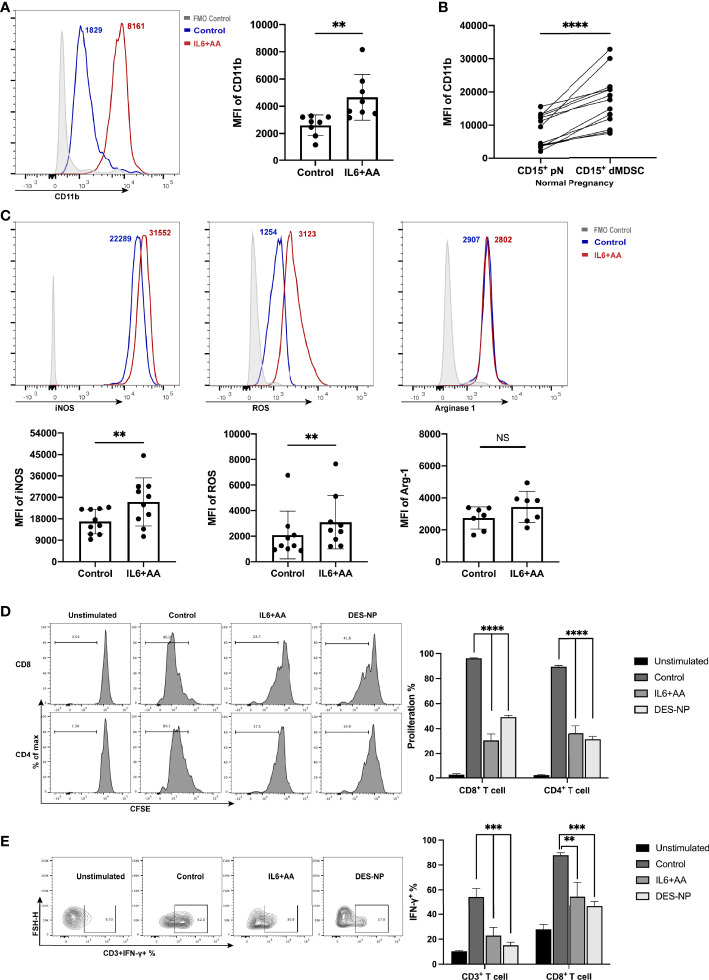
Immunosuppressive induction of circulating neutrophils after IL6 + AA treatment. **(A)** Flow cytometry was used to detect the expression of CD11b on neutrophils from healthy donors after stimulation with IL6 and AA (n = 8). **(B)** The expression of CD11b in CD15+ dMDSCs and autologous pN (n = 13). **(C)** The expression of immunosuppressive regulatory molecules, including iNOS (n = 10), ROS (n = 9), and ARG1 (n = 7), in neutrophils from healthy donors after IL6 + AA treatment. Neutrophils were previously treated with control medium, IL6+AA, or 50% (v/v) DES for 16 h. CD3/CD28-stimulated T cells were cocultured with treated neutrophils at a ratio of 2:1 for 3.5 days. Functional characteristics were then evaluated through the proportion of proliferative CD4+ T cells or CD8+ T cells (n = 6) **(D)** and the percentage of IFN-γ-expressing CD3+ T cells or CD8+ T cells (n = 6). **(E)** All data are represented as mean ± SD. Significance was determined by the unpaired Student’s t-test or by one-way ANOVA with Tukey’s post-hoc test. ****p < 0.0001, ***p < 0.001, **p < 0.01, NS, not significant. FMO, fluorescent minus one.

Consistent with the intracellular lipid accumulation, we also found that important immune regulatory molecules of PMN-MDSCs were upregulated in neutrophils after treatment with IL6 and AA. We found that IL6 + AA induced the significant upregulation of CD11b (*p* = 0.0071) (**Figures 5A**, **S4**), a phenotypic marker that is differentially expressed in decidual PMN-MDSCs, and autologous CD15^+^ neutrophils (**Figure 5B**). A range of immunosuppressive regulatory molecules are associated with the immune-regulatory activity of MDSCs, including iNOS, ROS, and ARG1 (49). IL6, along with supplementary AA, did not promote higher expression levels of ARG1 but did promote the expression of iNOS and ROS (**Figure 5C**; ARG1: *p* = 0.0802, iNOS: *p* = 0.0026, ROS: *p* = 0.0034). Similar changes were observed following induction with DES (**Figure S5**). Moreover, great importance should be given to T-cell suppression ability, as it defines the differentiation of neutrophils toward PMN-MDSCs. Consistent with the simulated decidua microenvironment, both CD4^+^ T-cell and CD8^+^ T-cell proliferations were notably suppressed in the presence of IL6 and AA (**Figures 5D**, **S6A**). Moreover, the IFN-γ secretion of CD3^+^ T cells and CD8^+^ T cells was also inhibited (**Figures 5E**, **S6B**).

**Figure 6:**
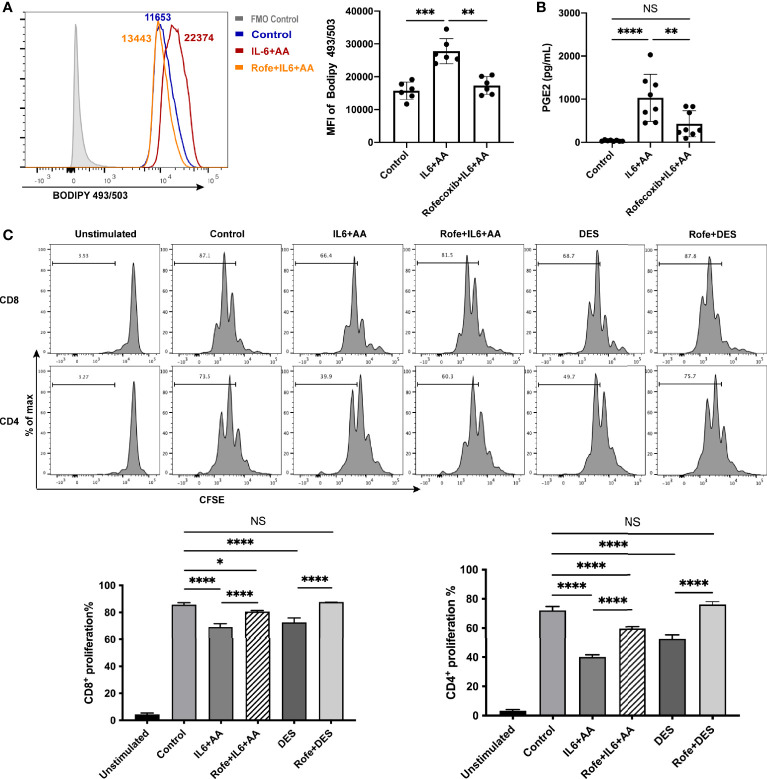
Arachidonic acid metabolism mediated intracellular lipid accumulation and the differentiation of PMN-MDSCs. **(A)** The increased lipid accumulation in circulating neutrophils stimulated by IL6+AA was blocked by a COX-2 inhibiter, rofecoxib (n = 6). **(B)** PGE2 secretion from the neutrophils of healthy donors was also inhibited by rofecoxib (n = 8). **(C)** Neutrophils were treated with control medium, IL6 + AA, IL6 + AA with rofecoxib, 50% (v/v) DES, or 50% (v/v) DES with rofecoxib for 16 h. Then, CD3/CD28-stimulated T cells were cocultured with treated neutrophils at a ratio of 2:1 for 3.5 days. The induced suppressive activity by IL6 + AA and DES were both blocked by rofecoxib (n = 6). All data are represented as mean ± SD. Significance was determined by one-way ANOVA with Tukey’s post-hoc test. ****p < 0.0001, ***p < 0.001, **p < 0.01, *p < 0.05. NS, not significant. FMO, fluorescent minus one.

To determine whether lipid-driven PMN-MDSC differentiation depends on AA metabolism, we next applied rofecoxib, a specific inhibitor of COX-2, and observed a significant reduction in lipid accumulation (**Figure 6A**). Data suggested that the blockade of AA metabolism exerted impact on the synthesis of intracellular lipid. Furthermore, IL6 with AA supplementation notably enhanced AA metabolism, as indicated by an increase in PGE2 synthesis; we also found that lower levels of PGE2 were associated with rofecoxib intervention (**Figure 6B**, **Figure S7**). In the presence of rofecoxib, the immunosuppressive activity induced by IL6 combined with AA or DES was disrupted (**Figure 6C**). Collectively, these data indicate that AA metabolism regulates intracellular lipid accumulation and the differentiation of PMN-MDSCs induced by the decidua microenvironment. In other words, IL6 is unable to generate PMN-MDSC-like neutrophils without the participation of AA and its metabolite PGE2.

### IL6 Stimulated STAT3 Phosphorylation and FABP5 Expression to Induce Decidual PMN-MDSCs

**Figure 7:**
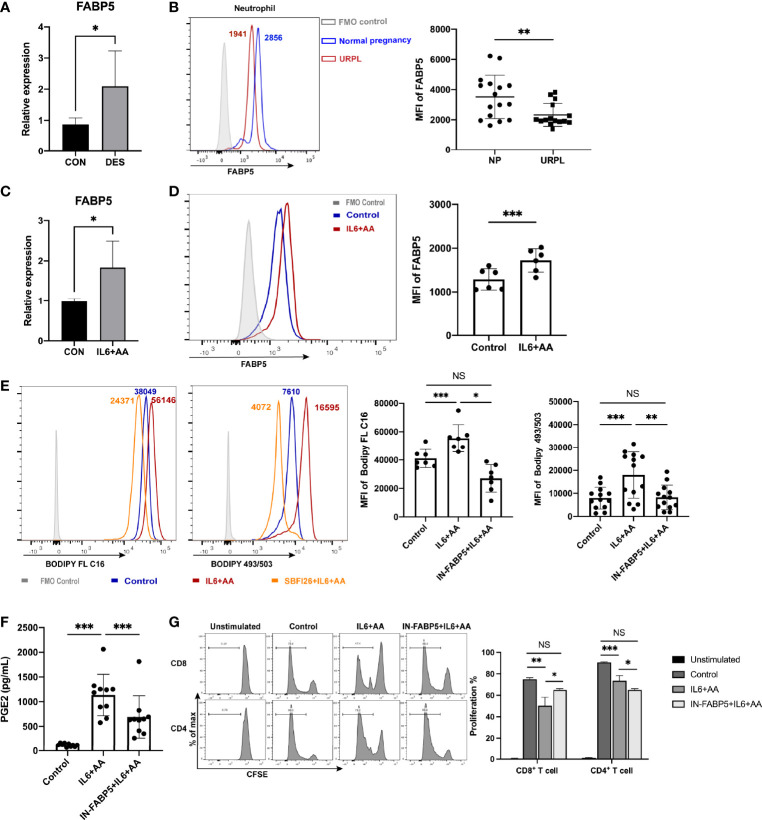
FABP5 regulated arachidonic acid metabolism and the differentiation of PMN-MDSCs via fatty acid uptake and lipid accumulation. **(A)** The upregulation of FABP5 after DES treatment was verified by qRT-PCR (n = 5). **(B)** FABP5 expression in circulating neutrophils between NP (n = 16) and URPL patients (n = 15) was evaluated by flow cytometry. Upregulated FABP5 expression in neutrophils after stimulation with IL6 + AA was tested by qRT-PCR (n = 5) **(C)** and flow cytometry (n = 6) **(D) (E)** Representative flow cytometry and summarized data show decreased fatty acid uptake (n = 7) and lipid accumulation (n = 13) in circulating neutrophils after FABP5 blockade. **(F)** The secretion of PGE2 stimulated by IL6 + AA was inhibited by SBFI26 (n = 10). **(G)** CD4+ and CD8+ T-cell proliferation were restored by SBFI26 (n = 6). All data are represented as mean ± SD. Significance was determined by unpaired Student’s t-test or one-way ANOVA with Tukey’s post-hoc test. ***p < 0.001, **p < 0.01, *p < 0.05, NS, not significant. FMO, fluorescent minus one.

The mRNA level of *FABP5* was significantly upregulated in DES-conditioned neutrophils; these findings were also verified by qRT-PCR (**Figure 7A**). Notably, we detected a significant difference in the FABP5 expression of circulating neutrophils when compared between normal pregnancies and URPL patients (**Figures 7B**, **S8**). This indicated that a deficiency in fatty acid transport may be associated with the impaired differentiation of decidual PMN-MDSCs in URPL, possibly due to the lower expression levels of FABP5 of peripheral neutrophils. A conspicuous upregulation in the mRNA level of *FABP5* was also observed in neutrophils when treated with IL6 and AA (**Figure 7C**). Flow cytometry further indicated a significant increase in the expression levels of FABP5 in the differentiation of neutrophils induced by IL6 + AA (**Figure 7D**). It appeared that IL6 and AA exerted a synergetic effect on the upregulation of FABP5. We then treated the peripheral CD15^+^ neutrophils with SBFI26, an inhibitor of FABP5 (50, 51). Both fatty acid uptake and subsequent intracellular lipid accumulation were significantly decreased owing to blockade of the pivotal fatty acid transport regulator FABP5 (**Figure 7E**). Furthermore, AA metabolism and the differentiation into PMN-MDSCs were significantly blocked; this was characterized by reduced PGE2 production and impaired T-cell suppression (**Figures 7F, G** **)**. Therefore, the functional regulation of FABP5 highlights the momentous driving effect of intracellular lipid on inducing AA metabolism and the differentiation of PMN-MDSCs.

**Figure 8:**
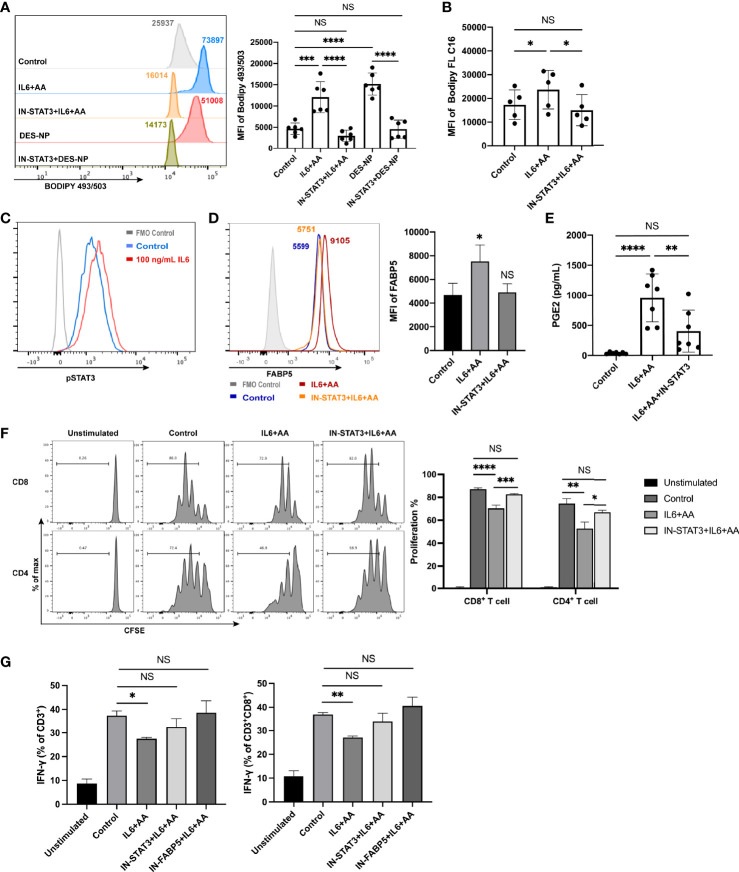
IL6 promoted the differentiation of PMN-MDSCs via STAT3 phosphorylation. **(A)** Lipid accumulation in circulating neutrophils from healthy donors treated with control medium, IL6 + AA, IL6 + AA with STAT3 inhibitor, 50% (v/v) DES, or 50% (v/v) DES with STAT3 inhibitor (n = 6). **(B)** The uptake of fatty acid in circulating neutrophils treated with control medium, IL6 + AA, or IL6 + AA with STAT3 inhibitor was evaluated by flow cytometry C16 (n = 5). **(C)** IL6 stimulated the STAT3 phosphorylation in neutrophils. **(D)** The upregulation of FABP5 expression stimulated by IL6 + AA was blocked by a STAT3 inhibitor (n = 5). **(E)** The secretion of PGE2 stimulated by IL6 + AA was inhibited after STAT3 blockade (n = 7). **(F)** Neutrophils were previously treated with control medium, IL6 + AA, and IL6 + AA with a STAT3 inhibitor for 16 h. Then, CD3/CD28-stimulated T cells were cocultured with treated neutrophils at a ratio of 2:1 for 3.5 days. The proliferation of CD4+ T cells or CD8+ T cells was restored after STAT3 blockade (n = 6). **(G)** The ability to produce IFN-γ in CD3+ T cells or CD8+ T cells increased under the action of a STAT3 or FABP5 inhibitor (n = 6). All data are represented as mean ± SD. Significance was determined by one-way ANOVA with Tukey’s post-hoc test. ****p < 0.0001, ***p < 0.001, **p < 0.01, *p < 0.05, NS, not significant. FMO, fluorescent minus one.

STAT3 and STAT5 are prominent transcription factors involved in the development of MDSCs (52, 53). We investigated whether the phosphorylation of STAT3 or STAT5 exerted influence on metabolic reprograming. As a result, Stattic, a STAT3 inhibitor, was found to significantly inhibit lipid accumulation while an inhibitor of STAT5 did not (**Figure S9**), thus implying that the activation of STAT3 is a key promotor of lipid metabolic reprograming for PMN-MDSC differentiation. STAT3 blockade also inhibited the IL6-mediated uptake of AA and intracellular lipid synthesis (**Figures 8A, B** **)**. Furthermore, IL6 was shown to remarkably activate STAT3, as previously reported (54) (**Figure 8C**). Hence, IL6 appears to stimulate STAT3 phosphorylation to induce intracellular lipid accumulation with the presence of arachidonic acid. The upregulation of FABP5 was also inhibited after STAT3 blockade (**Figure 8D**). Moreover, AA metabolism (the synthesis of PGE2) was notably impeded (**Figure 8E**). The blockade of STAT3 or FABP5 restored not only T-cell proliferation (**Figure 8F**) but also the attenuated secretion of IFN-γ (**Figure 8G**). To summarize, lipid accumulation drives the differentiation of neutrophils into PMN-MDSCs when induced by decidua-derived IL6 and AA; this mechanism occurred *via* the pSTAT3/FABP5/PGE2 pathway.

## Discussion

Lipid plays a critical role in early pregnancy. Abnormal lipid metabolism impairs endometrial receptivity and early embryo implantation (55–58) and can potentially become a risk factor for URPL (59–61). With the comprehensive investigation of various cells at the maternal–fetal interface, the impact of lipid metabolism in successful pregnancies is becoming increasingly prominent. The decidualization of endometrial stromal cells is highly dependent on both glycolysis and the utilization of lipid *via* fatty acid beta-oxidation (62). The development of extravillous trophoblast is also accompanied by the accumulation of glycogen and intracellular lipid (63). Furthermore, the deregulation of lipid metabolism is associated with abnormal placental development and early miscarriage (60). MDSCs are important immune regulators during early pregnancy (11–14). Successful implantation and pregnancy are inseparable from the expansion and enrichment of MDSCs (7, 11, 12, 14, 64, 65). In the present study, we identified the significance of intracellular lipid accumulation to decidual PMN-MDSCs. Compared with normal pregnancies, URPL patients exhibited significantly less intracellular lipid content in PMN-MDSCs, along with a reduction in the proportion of PMN-MDSCs. This positive correlation suggests that impaired lipid synthesis may lead to insufficient decidual PMN-MDSC differentiation in URPL.

Recent studies have indicated that lipid can modulate the function of MDSCs, in addition to maintaining cell membrane integrity, homeostasis, signaling, and health (35, 66). Lipid metabolism is involved in regulating the differentiation and function of immune cells (34, 40, 67–69). The promotion of M2 macrophages that dominate the maternal–fetal tolerance is accompanied by fatty acid oxidization, thus suggesting the participation of lipid in the differentiation of immune cells that occur in human pregnancy (70). In a previous study, we showed that the decidua microenvironment can endow circulating neutrophils with an immunosuppressive function and phenotype (20). In the present study, we detected more lipid accumulation and enrichment of the fatty acid metabolic pathways in decidual PMN-MDSCs than in autologous neutrophils. Therefore, we hypothesize that intracellular lipid and its metabolism are involved in regulating the differentiation and function of PMN-MDSCs during early pregnancy. Increased lipid accumulation in tumor-infiltrating MDSCs is known to be accompanied by enhanced immunosuppressive activity (37, 39). It has been shown that MDSCs exhibit increased levels of fatty acid uptake (37), lipid accumulation (39, 66), and fatty acid oxidation (27, 35, 37) to support their suppressive function. In the current study, we observed that the decidua microenvironment promoted fatty acid uptake and lipid accumulation in circulating neutrophils. Moreover, inhibition of the T-cell response, defined as the hallmark of MDSCs, further suggests that increased lipid levels can activate the immunoregulatory mechanisms and facilitate immune tolerance. Once lipid accumulation becomes dysfunctional, the activity of T-cell proliferation will be restored. Furthermore, we found that the levels of CD11b in neutrophils were upregulated after treatment; CD11b-dependent delivery into immunological synapses has been reported to mediate the negative immunoregulatory responses (71). We also found that intracellular lipid accumulation also upregulated the expression of the immunoregulatory factors, iNOS and ROS, and that this was associated with the inhibitory activity of MDSCs. Thus, we demonstrated that intracellular lipid promote the differentiation of neutrophils into PMN-MDSCSs *via* the induction of immunosuppressive functionality.

FABP5 belongs to the family of FABPs that coordinates lipid trafficking and response with cells. FABP5 acts as an intracellular chaperone that can bind and transport fatty acids for subsequent signal transduction (72, 73). It has been reported that FABP5 can reprogram lipid metabolism and promote progression and metastasis in tumors (74–76). Furthermore, FABP5 has been shown to act as a critical regulator of immune cells in microenvironments *via* lipid metabolism, including the protection of tissue-resident memory T cells (77), the induction of suppressive T regulatory cells (67, 78), the maintenance of T lymphocyte homeostasis (79), the production of tolerogenic plasmacytoid dendritic cells (78), and macrophage polarization, and has also been identified as a prognostic immune-metabolic marker (80, 81). Similarly, we identified the immunomodulatory effect of FABP5 during the differentiation of decidual PMN-MDSCs. The expression of FABP5 in neutrophils was upregulated after exposure to decidua-derived factors. In addition, FABP5 was shown to mediate AA uptake, intracellular lipid accumulation, and PGE2 synthesis, thus contributing to the differentiation of neutrophils into PMN-MDSCs. The induced T-cell inhibition disappeared following the application of the FABP5 inhibitor SBFI26. Similar observations in MDSCs have also been reported recently with regard to other proteins related to fatty acid transport. The genetic depletion of fatty acid translocase CD36 was previously shown to inhibit the immunomodulatory function of tumor-infiltrating MDSCs (37). Fatty acid transport 2 (FATP2) reprograms neutrophils; the selective inhibition of FATP2 has been shown to abrogate the activity of MDSCs in cancer (39). Together with our current findings, these previous data support the significance of lipid metabolic and functional reprogramming in the plasticity of tissue MDSCs. The STAT family is known to regulate the function of MDSCs by coordinating various activities; STAT3 signaling is crucial for the activation and differentiation of MDSCs (25). We found that the expression levels of FABP5 in neutrophils were mediated by STAT3 phosphorylation. Interestingly, we also detected lower levels of FABP5 expression in the neutrophils of URPL women, thus implying its potential value as a marker of miscarriage in future studies.

PGE2 has emerged as a key molecule in the biology of MDSCs; it has been shown to promote the development and induction of MDSCs and participate in the suppressive activity (39, 45, 47). Moreover, PGE2 is thought to exert critical effects on the promotion of endometrial receptivity and decidualization (82, 83), and the modulation of uterine contractility during pregnancy (83, 84). As a metabolite of AA metabolism, the secretion of PGE2 from decidual PMN-MDSCs in URPL was significantly lower than that in normal pregnancies, thus highlighting the role of AA metabolism in the promotion of PMN-MDSC differentiation. However, the amount of AA did not differ significantly in groups. A previous metabonomic analysis also indicated that the levels of the poly-unsaturated fatty acid AA in the decidua and placenta were similar when compared between spontaneous pregnancy loss and normal pregnancy, although a variety of other fatty acids did show differences (59). The regulation of AA uptake and lipid accumulation, but not the amount of AA within the microenvironment, appear to be more important for the differentiation of decidual PMN-MDSCs. These results indicate the importance of the pSTAT3/FABP5/PGE2 axis in driving lipid metabolism and function during the differentiation of neutrophils into PMN-MDSCs.

IL6 is an inflammatory cytokine that can exert pleiotropic functions. The IL-6R complex is activated by binding with gp130 and IL-6R to then stimulate JAK and STAT signaling (54). IL6 plays a strong role in the accumulation and activation of MDSCs in various pathogenic conditions (54, 85, 86). The levels of IL6 are also known to increase during pregnancy, and the dysregulation of IL6 may disturb implantation and be harmful to the success of pregnancy (86, 87). Consistent with our present study, reduction in the levels of IL6 was also observed in women undergoing abortion in previous studies (87–89). In the present study, we found that IL6 regulated the FABP5 expression to induce AA uptake, lipid accumulation, and PGE2 synthesis when AA is supplemented in the cultivation system and that this occurred *via* the activation of STAT3. IL6 can exert a synergetic effect with the polyunsaturated fatty acids within the decidua microenvironment. Therefore, our findings indicate that decidua-derived IL6 can facilitate the differentiation of circulating neutrophils into PMN-MDSCs by mediating AA metabolism.

In the present study, we focused on the regulatory effects of intracellular lipid on MDSCs and provide an enhanced understanding of immune-metabolic reprogramming during pregnancy. Previous studies have only reported the function of GM-CSF; here, we propose that IL6 can induce neutrophils to differentiate toward PMN-MDSCs *via* promoting the utilization of arachidonic acid within the decidua microenvironment, thus providing a rationale for immunotherapy using IL6. Our study was only based on *in vitro* experiments; therefore, further studies in pregnant mice may provide us with in-depth information.

**Figure 9:**
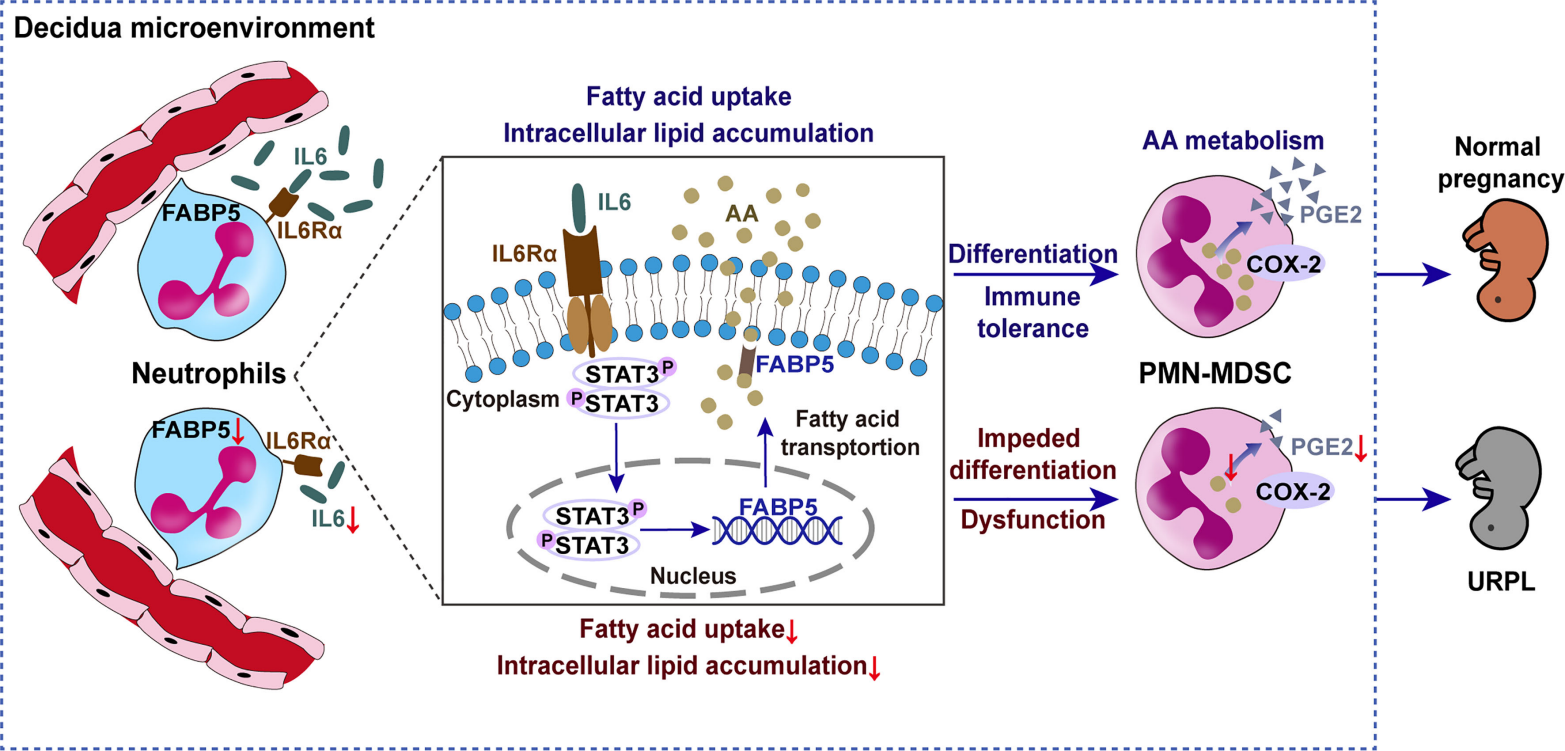
Schematic diagram showing the impact of lipid metabolism on the differentiation of decidual PMN-MDSCs between NP and URPL. Decidual-derived IL6 induced arachidonic acid uptake and intracellular lipid accumulation and then promoted PGE2 synthesis and immunosuppressive PMN-MDSC differentiation in the decidua via pSTAT3/FABP5/COX-2/PGE2 signaling. Factors resulting in lipid metabolism dysfunction can lead to impaired PMN-MDSCs differentiation and function in URPL.

In summary, intracellular lipids drive decidual PMN-MDSC differentiation *via* AA metabolism. Circulating neutrophils undergo increased fatty acid uptake and lipid accumulation when induced by decidua-derived IL6, thus acquiring a PMN-MDSC-like phenotype and function; our findings indicate that this occurs *via* pSTAT3/FABP5/PGE2 signaling. The differences of intracellular lipid and related key factors between URPL and normal pregnancy are likely to explain the impaired differentiation of PMN-MDSCs in URPL (**Figure 9**). These findings enrich our understanding of the physiological mechanisms of immune tolerance during pregnancy and the pathological mechanisms of immune disorders in URPL. Our data indicate the potential protective role of IL6 in pregnancy complications and the possible use of FABP5 as a marker in peripheral neutrophils.

## Data Availability Statement

The datasets presented in this study can be found in online repositories. The names of the repository and accession numbers can be found as follows: Gene Expression Omnibus - GSE19285 and GSE193491.

## Ethics Statement

The studies involving human participants were reviewed and approved by the Human Research Ethics Committee of Renji Hospital. The patients/participants provided their written informed consent to participate in this study.

## Author Contributions

QW, XyZ, and AZ designed the study. QW, XyZ, CL, MX, and WB carried out the experiments. XyZ, MX, SS, CC, and ML coordinated the sample collection and performed the literature search. QW, XyZ, and CL analyzed the data and prepared the figures. QW completed the manuscript. XxZ provided technical support. AZ revised the manuscript. All authors contributed to the article and approved the submitted version.

## Funding

This study was supported by the National Natural Science Foundation of China (No. 82071725) and the National Natural Science Foundation of China (No. 81871179).

## Conflict of Interest

The authors declare that the research was conducted in the absence of any commercial or financial relationships that could be construed as a potential conflict of interest.

## Publisher’s Note

All claims expressed in this article are solely those of the authors and do not necessarily represent those of their affiliated organizations, or those of the publisher, the editors and the reviewers. Any product that may be evaluated in this article, or claim that may be made by its manufacturer, is not guaranteed or endorsed by the publisher.
