# Supplementary figures and images for: Intracellular Lipid Accumulation Drives the Differentiation of Decidual Polymorphonuclear Myeloid-Derived Suppressor Cells via Arachidonic Acid Metabolism
Source: Front Immunol. 2022 May 18;13:868669. doi: 10.3389/fimmu.2022.868669 (PMC9159278; doi:10.3389/fimmu.2022.868669)

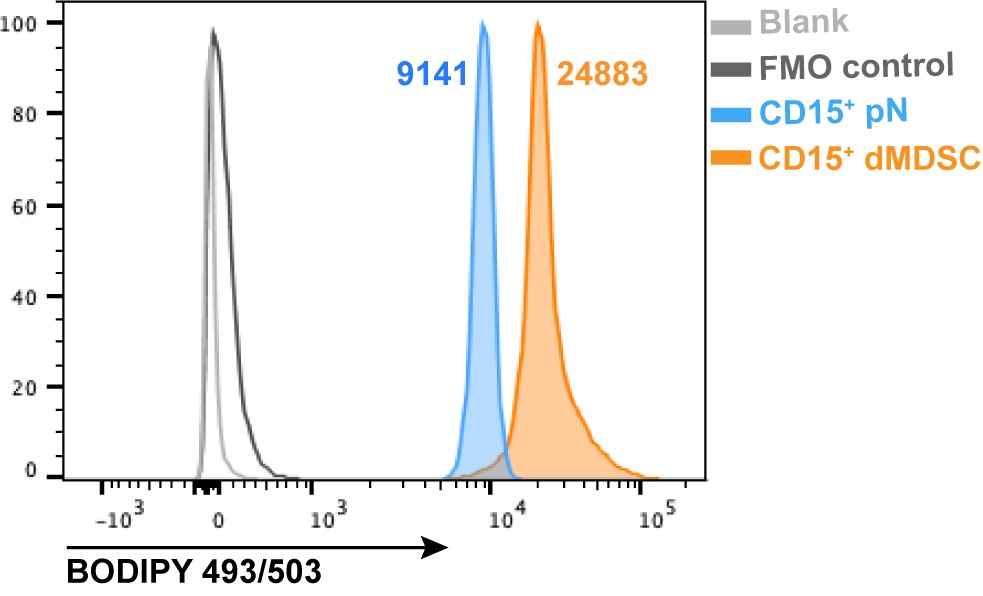

Supplement: Supplementary file 1 [file Image_1.tif]

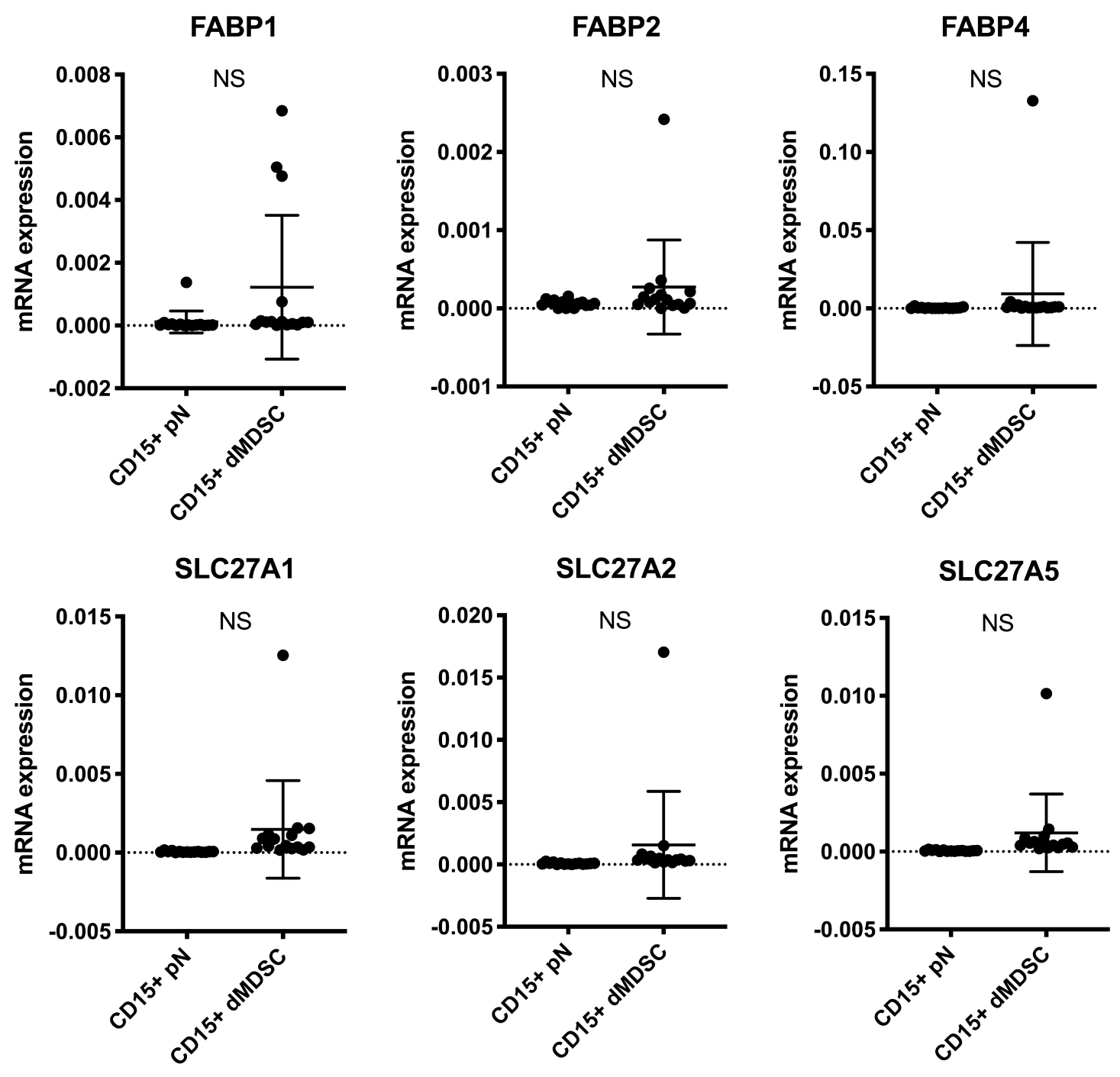

Supplement: Supplementary file 2 [file Image_2.tif]

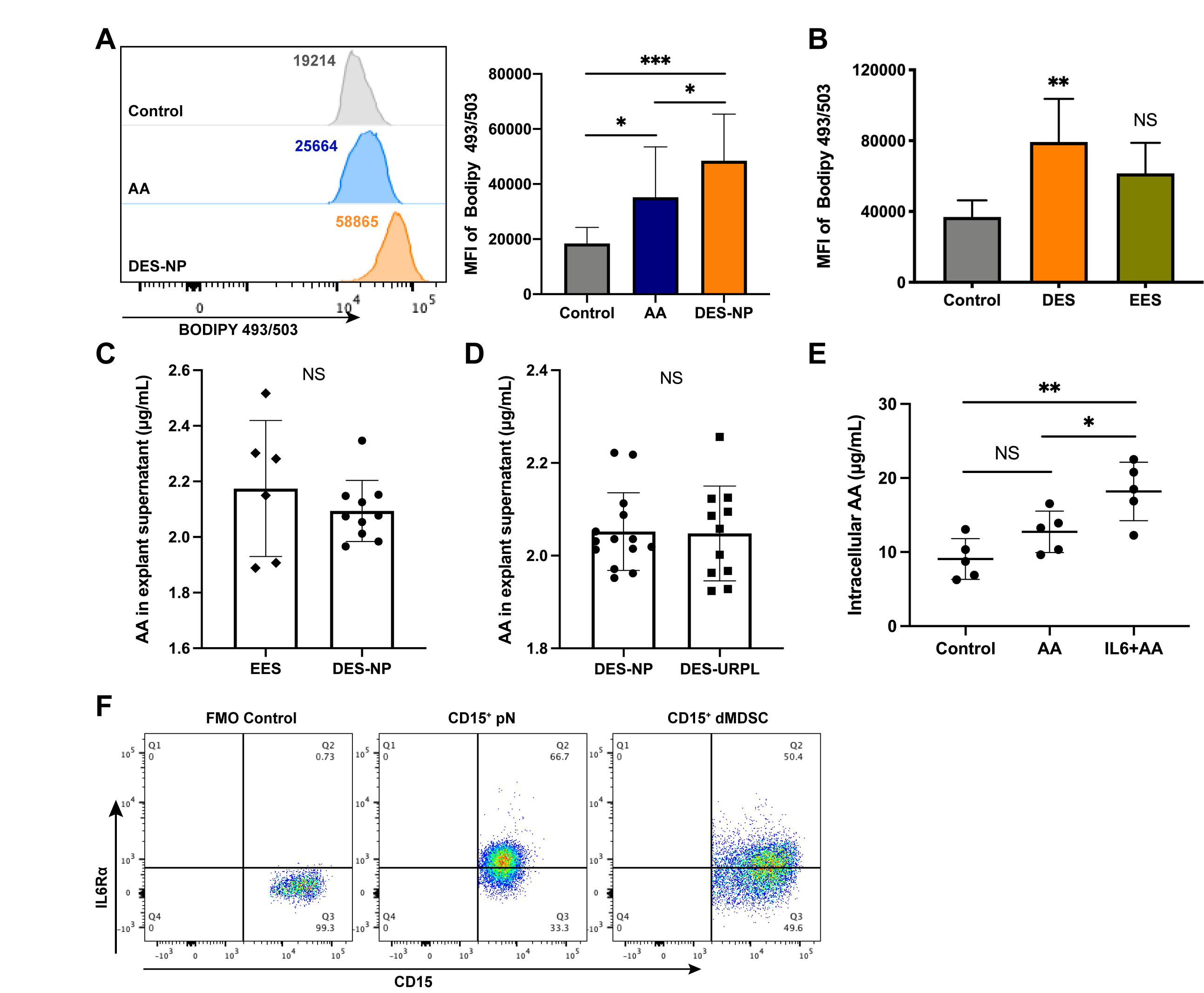

Supplement: Supplementary file 3 [file Image_3.tif]

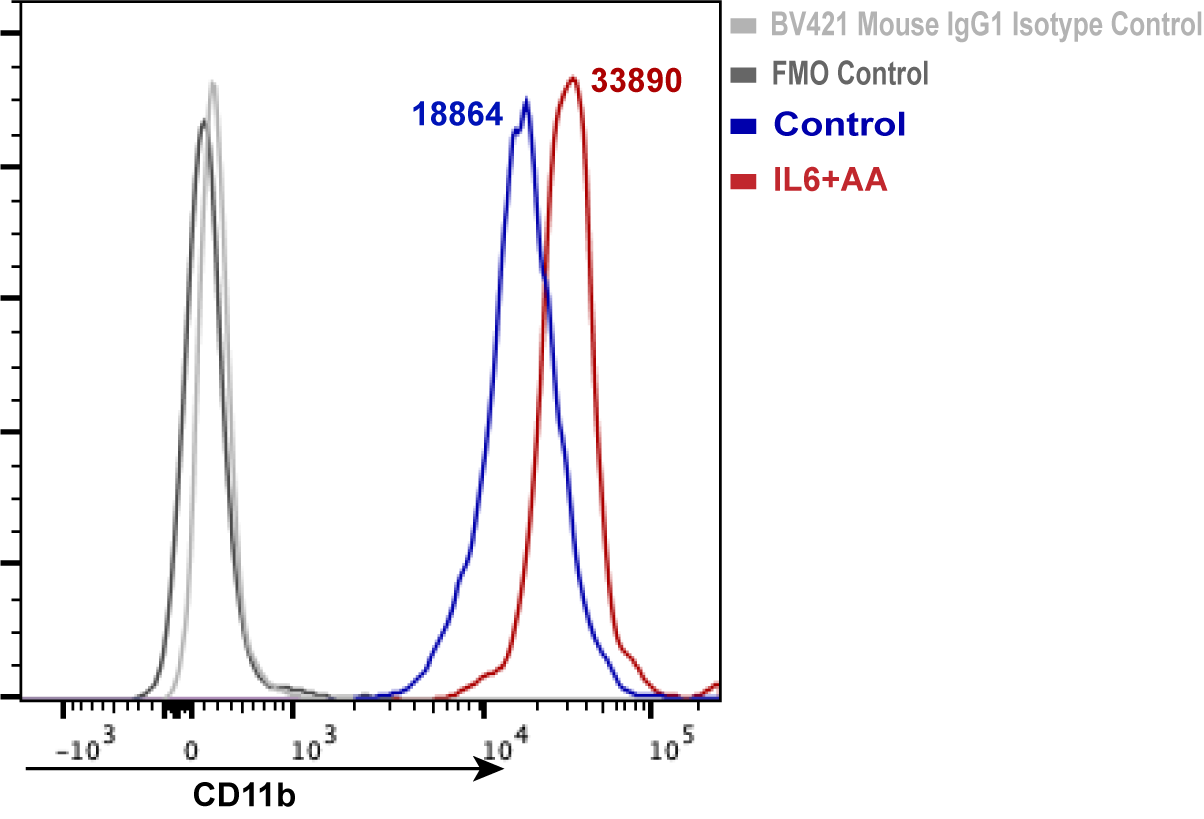

Supplement: Supplementary file 4 [file Image_4.tif]

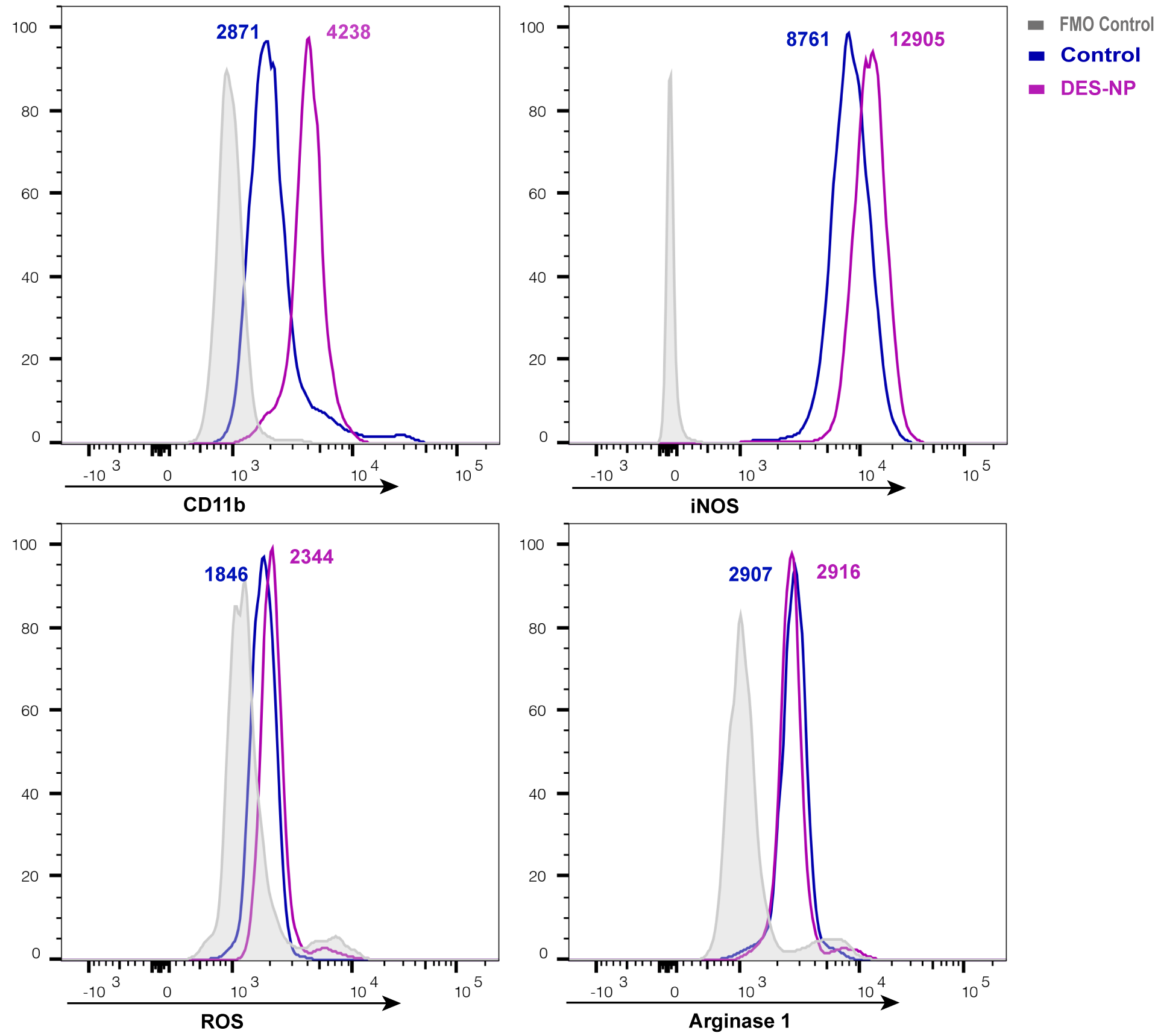

Supplement: Supplementary file 5 [file Image_5.tif]

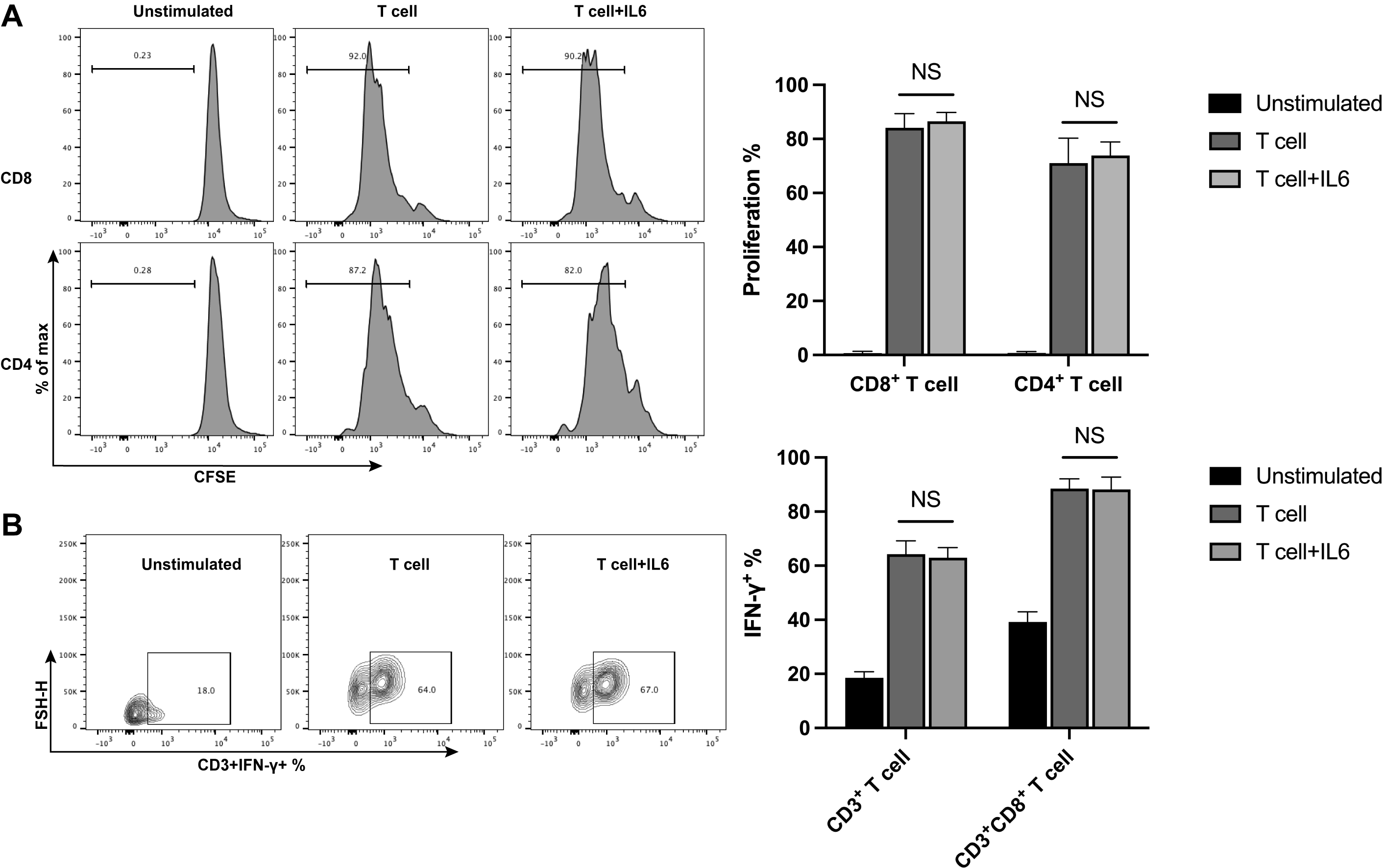

Supplement: Supplementary file 6 [file Image_6.tif]

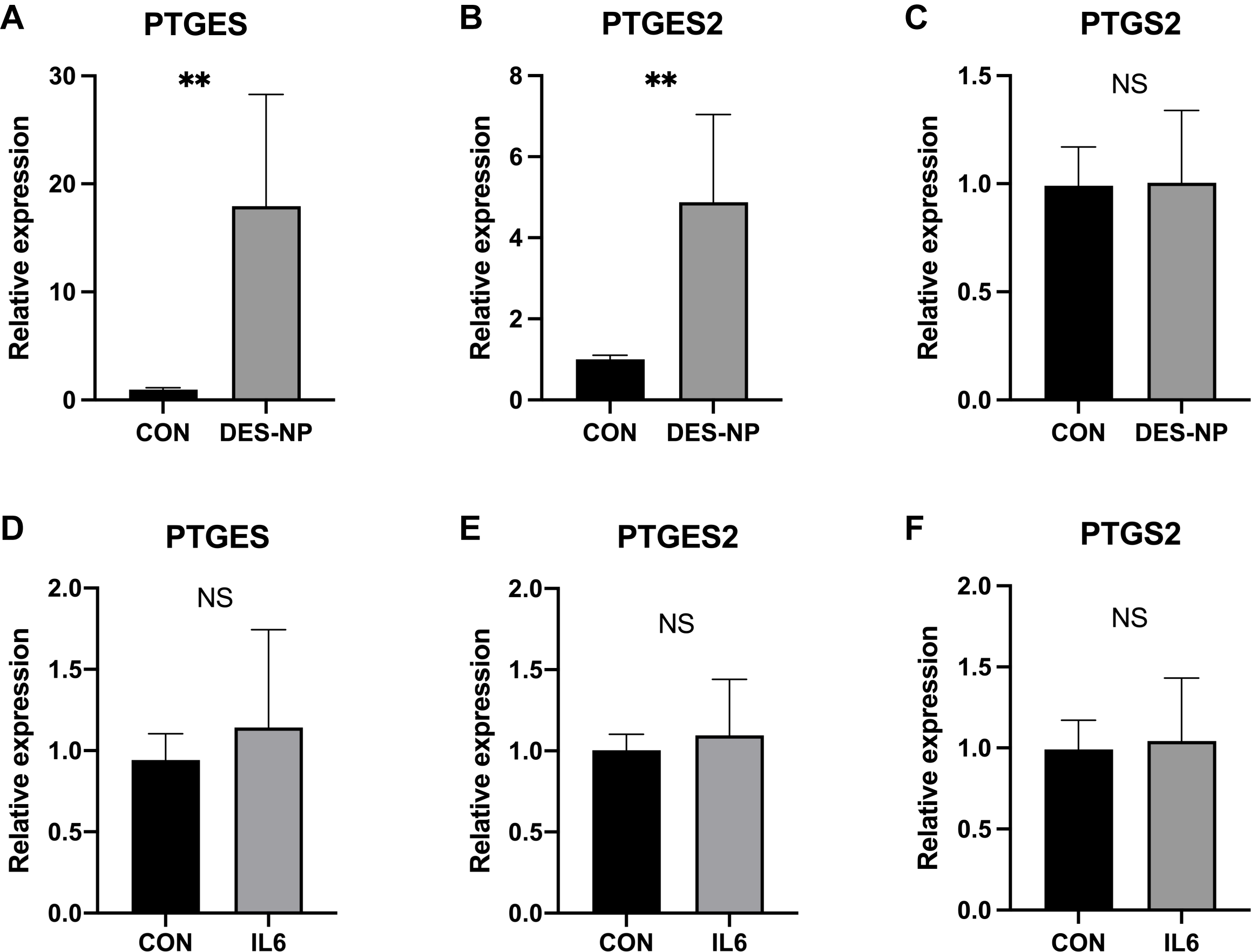

Supplement: Supplementary file 7 [file Image_7.tif]

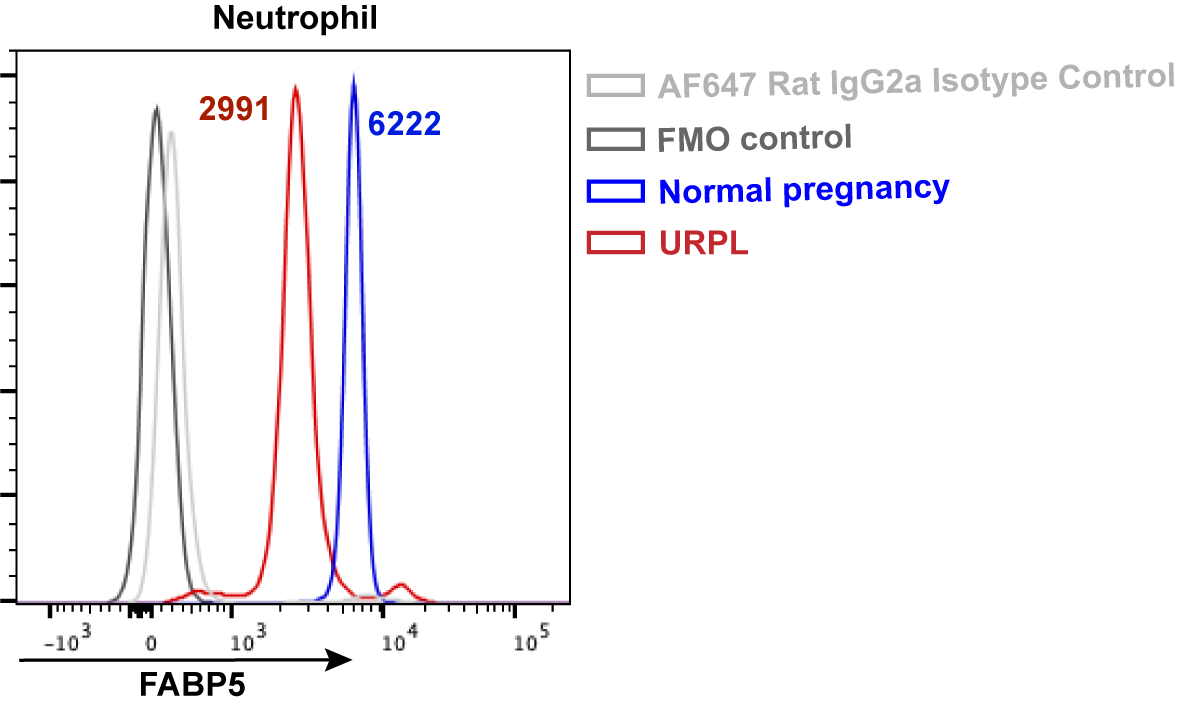

Supplement: Supplementary file 8 [file Image_8.tif]

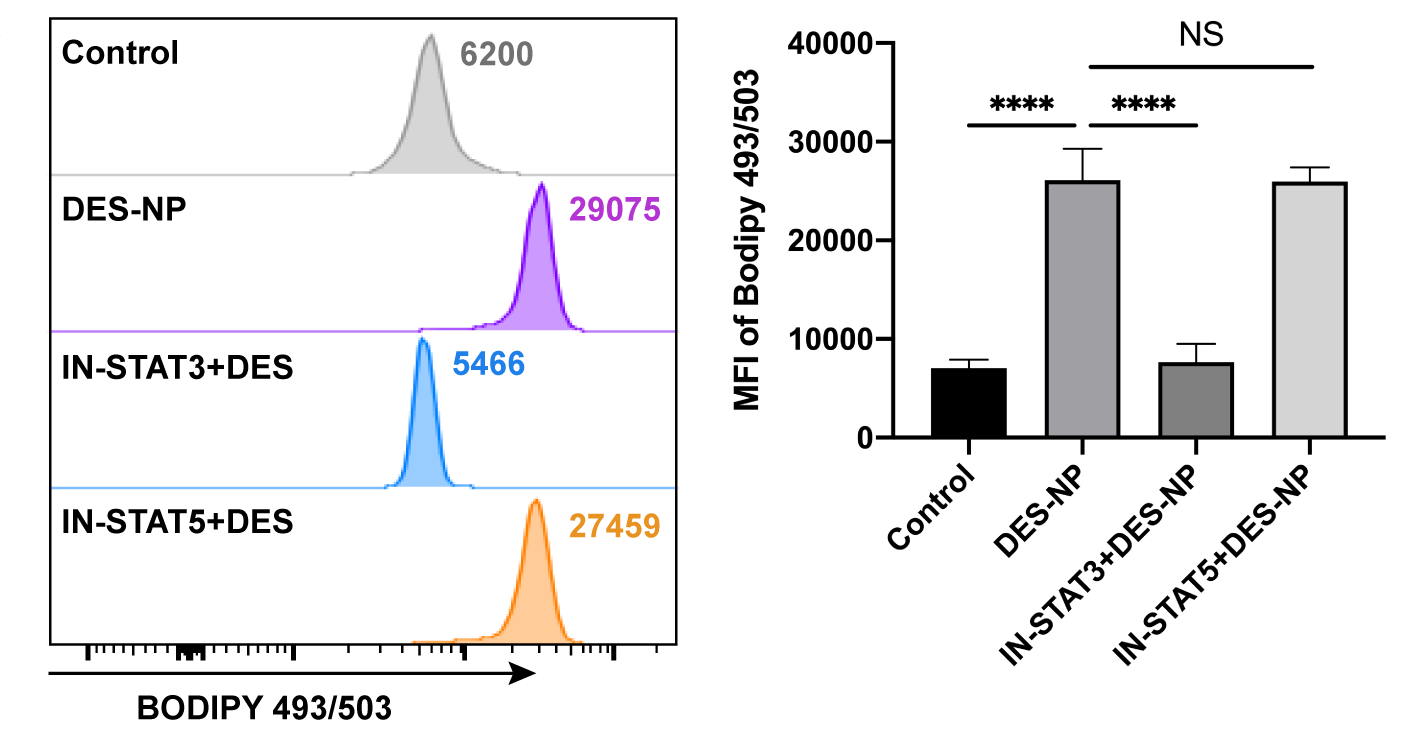

Supplement: Supplementary file 9 [file Image_9.tif]
