# Supplementary material for: Intracellular Lipid Accumulation Drives the Differentiation of Decidual Polymorphonuclear Myeloid-Derived Suppressor Cells via Arachidonic Acid Metabolism
Source: Front Immunol. 2022 May 18;13:868669. doi: 10.3389/fimmu.2022.868669 (PMC9159278; doi:10.3389/fimmu.2022.868669)
Supplement: Supplementary file 10 [file DataSheet_1.docx]

**Supplementary Figure S1. The gating control of BODIPY 493/503.**

Cells without staining (Blank) and FMO control were used as gating controls. FMO, Fluorescent minus one.

**Supplementary Figure S****2. Expression of lipid transport receptors between CD15^+^ dMDSCs and autologous pN.**

There was no difference in *FABP1*, *FABP2*, *FABP4*, *SLC27A1*, *SLC27A2* and *SLC27A5* between CD15^+^ dM and pN in normal pregnancies. All data are represented as mean ± SD. Significance was determined by paired Student’s *t*-test. NS, not significant.

**Supplementary Figure S3. Exploration of the vital molecules regulating intracellular lipid accumulation.**

(**A**) Lipid accumulation of neutrophils after treatment of DES or arachidonic acid (AA) (n=6). (**B**) Lipid accumulation of neutrophils treated with DES in comparison with endometrial explant supernatant (EES) (n=5). (**C**) and (**D**) There was no difference in AA either between EES (n=6) and DES (n=10), or between DES-NP (n=14) and DES-URPL (n=11), as detected by ELISA. (**E**) ELISA assay determined that IL6 upregulated levels of intracellular AA of neutrophils (n=5). (**F**)Expression of IL6-Rα on CD15^+^ dMDSCs and pN. All data are represented as mean ± SD. Significance was determined by one-way ANOVA with Tukey’s post hoc test. ****p*<0.001, **p*<0.05, NS, not significant. FMO, Fluorescent minus one.

**Supplementary Figure S4. The gating control of CD11b.**

Both isotype (BV421 Mouse IgG1) and FMO control were used as gating controls. FMO, Fluorescent minus one.

**Supplementary Figure S5. Expression of regulatory markers after DES treatment.**

The expression of CD11b, iNOS, ROS was upregulated while ARG1 was not after the induction of DES.

**Supplementary Figure S6. Effects of IL6 alone on T cells.**

IL-6 alone did not affect the proliferation of CD4^+^ T cells or CD8^+^ T cells (**A**) and the ability to produce IFN-γ in CD3^+^ T cells or CD8^+^ T cells (**B**). All data are represented as mean ± SD. Significance was determined by one-way ANOVA with Tukey’s post hoc test. NS, not significant.

**Supplementary Figure S7.** **Effects of DES and IL6 alone on the expression of main inducible PGE2 synthetic enzymes****.**

DES upregulated the expression of *PTGES* (n=6) (**A**), *PTGES2* (n=6) (**B**), and did not affect the expression of *PTGS2* (n=6) (**C**). IL-6 alone did not affect the expression of *PTGES* (n=6) (**D**), *PTGES2* (n=6) (**E**), and *PTGS2* (n=6) (**F**). All data are represented as mean ± SD. Significance was determined by the unpaired Student’s *t*-test. ***p*<0.01, NS, not significant.

**Supplementary Figure S8. The gating control of FABP5.**

Both isotype (AF647 Rat IgG2a) and FMO control were used as gating controls. FMO, Fluorescent minus one.

**Supplementary Figure S9. Effect of a STAT3 or STAT5 inhibitor on increased lipid accumulation after the induction of DES.**

Lipid accumulation was detected in DES-conditioned neutrophils with the intervention of a STAT3 or STAT5 inhibitor by flow cytometry (n=5). All data are represented as mean ± SD. Significance was determined by one-way ANOVA with Tukey’s post hoc test. *****p*<0.0001, NS, not significant.

**Supplemental Table S1. Clinical characteristics of women included in lipid accumulation of decidual PMN-MDSCs and autologous neutrophils**

|  | **NP (n=61)** |
| --- | --- |
| Age (years) | 31.57±4.83 |
| Gestation age (days) | 53.16±6.79 |
| Gravidity | 2-7 |
| Parity | 1-3 |
| Previous elective termination | 0-5 |

NP: normal pregnancy

**Supplemental Table S2. Clinical characteristics of women included in lipid accumulation of decidual PMN-MDSCs between NP and URPL patients**

|  | **NP (n=21)** | **URPL (n=21)** | ***P*** |
| --- | --- | --- | --- |
| Age (years) | 32.67±4.81 | 33.19±4.62 | 0.7217^a^ |
| Gestation age (days) | 56.24±7.42 | 60.29±5.78 | 0.0556^a^ |
| Previous pregnancy loss | 0 | 2-5 | - |
| Parity | 1-3 | 0 | - |

NP: normal pregnancy

URPL: unexplained recurrent pregnancy loss

^a^ Unpaired Student’s t test.

**Supplemental Table S3. Primers used for quantitative real-time PCR**

| **Genes** | **Primers** |
| --- | --- |
| *CD36* | Forward: 5’ CTGTTATGGGGCTATAGGGATC 3’ |
|  | Reverse: 5’ ACTCCATCTGCAGTATTGTTGT 3’ |
| *MSR1* | Forward: 5’ CAGGAAATTCTGGACCAAAAGG 3’ |
|  | Reverse: 5’ CAGCGATCGTCACAAATTGTAC 3’ |
| *FABP1* | Forward: 5’ GTCCAAAGTGATCCAAAACGAA 3’ |
|  | Reverse: 5’ CGGTCACAGACTTGATGTTTTT 3’ |
| *FABP2* | Forward: 5’ GTTTTTGAACTTGGTGTCACCT 3’ |
|  | Reverse: 5’ GTCTGTCCGTTTGAATTTTCCA 3’ |
| *FABP3* | Forward: 5’ CTAGTGGACAGCAAGAATTTCG 3’ |
|  | Reverse: 5’ AGCTGATCTCTGTGTTCTTGAA 3’ |
| *FABP4* | Forward: 5’ GGCCAAACCTAACATGATCATC 3’ |
|  | Reverse: 5’ TTATGGTGCTCTTGACTTTCCT 3’ |
| *FABP5* | Forward: 5’ CCAAGCCAGATTGTATCATCAC 3’ |
|  | Reverse: 5’ CATCAGCTGTGGTTTCTTCAAA 3’ |
| *FABP6* | Forward: 5’ CTGCAGAGAATGAAACAGACAC 3’ |
|  | Reverse: 5’ AAGGAGCTTCATGAACTCATCA 3’ |
| *SLC27A1* | Forward: 5’ GGTCGGCTCCTGTGGTTTCAAC 3’ |
|  | Reverse: 5’ AGCAGCTCCATTGTGTCCTCATTG 3’ |
| *SLC27A2* | Forward: 5’ GTGCCTCAATTACAACATCCG 3’ |
|  | Reverse: 5’ TAAGGCTTGGCAGTATCTCTTC 3’ |
| *SLC27A3* | Forward: 5’ CCCTGCTGGAATTAGCGATTT 3’ |
|  | Reverse: 5’ GGGCGAGGTAGATCACATCTT 3’ |
| *SLC27A4* | Forward: 5’ GTACTCAAGCAGTGTAGCCAAC 3’ |
|  | Reverse: 5’ CTCATTGCGGTTCTCCATGAA 3’ |
| *SLC27A5* | Forward: 5’ CACGTTCAAACTGATGAAGACC 3’ |
|  | Reverse: 5’ CCAGTACAAACAGAGGGTCAA 3’ |
| *SLC27A6* | Forward: 5’ TCATAGTTCAGCAGCTATCCTG 3’ |
|  | Reverse: 5’ ATACTTCTTGCAGTCACTCCAA 3’ |
| *PTGES* | Forward: 5’ TCCTAACCCTTTTGTCGCCTG 3’ |
|  | Reverse: 5’ CGCTTCCCAGAGGATCTGC 3’ |
| *PTGES2* | Forward: 5’ GCGGCCATGTACCTCATCAG 3’ |
|  | Reverse: 5’ AAATCAGCGAGATTCGGCTTC 3’ |
| *PTGS2* | Forward: 5’ CTGGCGCTCAGCCATACAG 3’ |
|  | Reverse: 5’ CGCACTTATACTGGTCAAATCCC 3’ |
| *GAPDH* | Forward: 5’CAGGAGGCATTGCTGATGAT 3’ |
|  | Reverse: 5’GAAGGCTGGGGCTCATTT 3’ |
